# Bacterial interactions during sequential degradation of cyanobacterial necromass in a sulfidic arctic marine sediment

**DOI:** 10.1111/1462-2920.14297

**Published:** 2018-09-03

**Authors:** Albert L. Müller, Claus Pelikan, Julia R. de Rezende, Kenneth Wasmund, Martina Putz, Clemens Glombitza, Kasper U. Kjeldsen, Bo Barker Jørgensen, Alexander Loy

**Affiliations:** ^1^ Division of Microbial Ecology, Department of Microbiology and Ecosystem Science Research Network Chemistry meets Microbiology, University of Vienna Vienna Austria; ^2^ Austrian Polar Research Institute Vienna Austria; ^3^ Center for Geomicrobiology, Department of Bioscience Aarhus University Aarhus Denmark; ^4^ NASA Ames Research Center Moffett Field CA USA

## Abstract

Seafloor microorganisms impact global carbon cycling by mineralizing vast quantities of organic matter (OM) from pelagic primary production, which is predicted to increase in the Arctic because of diminishing sea ice cover. We studied microbial interspecies‐carbon‐flow during anaerobic OM degradation in arctic marine sediment using stable isotope probing. We supplemented sediment incubations with ^13^C‐labeled cyanobacterial necromass (spirulina), mimicking fresh OM input, or acetate, an important OM degradation intermediate and monitored sulfate reduction rates and concentrations of volatile fatty acids (VFAs) during substrate degradation. Sequential 16S rRNA gene and transcript amplicon sequencing and fluorescence *in situ* hybridization combined with Raman microspectroscopy revealed that only few bacterial species were the main degraders of ^13^C‐spirulina necromass. *Psychrilyobacter, Psychromonas, Marinifilum, Colwellia, Marinilabiaceae* and *Clostridiales* species were likely involved in the primary hydrolysis and fermentation of spirulina. VFAs, mainly acetate, produced from spirulina degradation were mineralized by sulfate‐reducing bacteria and an *Arcobacter* species. Cellular activity of *Desulfobacteraceae* and *Desulfobulbaceae* species during acetoclastic sulfate reduction was largely decoupled from relative 16S rRNA gene abundance shifts. Our findings provide new insights into the identities and physiological constraints that determine the population dynamics of key microorganisms during complex OM degradation in arctic marine sediments.© 2018 Society for Applied Microbiology and John Wiley & Sons Ltd

## Introduction

The Earth's seafloor is habitat for more than half of the microbial cells in the marine environment (Kallmeyer *et al*., 2012). Microorganisms in marine sediments are highly dependent on energy and carbon that is derived from the degradation of detrital organic matter (OM) (Jørgensen and Boetius, 2007), which is produced by marine phytoplankton (Arndt *et al*., 2013). Although only 1% of the OM exported from surface waters reaches the seafloor on a global scale (Hedges and Keil, 1995), regional differences are high and sediments in coastal regions receive approximately 6–12% of pelagic primary production (Dunne *et al*., 2007). The labile fraction of OM that reaches the seafloor is gradually degraded and mineralized by the concerted activity of diverse anaerobic microorganisms (Arndt *et al*., 2013; Orsi *et al*., 2018). Cellular macromolecules such as proteins, nucleic acids, lipids and polysaccharides that make‐up the majority of phytoplankton debris (Burdige, 2007) are degraded by mostly unknown microorganisms into oligomers and monomers, which are subsequently fermented to a range of products, including volatile fatty acids (VFAs), H_2_ and CO_2_ (Burdige, 2007; Muyzer and Stams, 2008). In continental shelf sediments, the most favorable electron acceptors (O_2_, Fe(III), Mn(IV), ${\text{NO}}_{3}^{-}$) are depleted first (Froelich *et al*., 1979) and sulfate reduction is the predominant process that accounts for the oxidation of half of the VFAs that are produced during anaerobic OM breakdown (Jørgensen, 1982). Thus, on a global scale sulfate‐reducing microorganisms (SRM) facilitate the remineralization of approximately 29% of the total OM flux to the entire seafloor (Bowles *et al*., 2014).

The Arctic is severely impacted by climate change, resulting in a constant decline of the arctic sea ice cover (Vaughan *et al*. 2013). This allows marine primary production to prevail (Arrigo *et al*., 2008) and predictions suggest this will initially cause an increased export of OM to the seafloor (Lalande *et al*., 2009; Sørensen *et al*., 2015). OM degradation rates and pathways in arctic marine sediments have been intensively studied in Svalbard fjords with a focus on enzymatic hydrolysis, sulfate reduction, iron reduction and denitrification (Arnosti and Jørgensen, 2006; Finke *et al*., 2007; Canion *et al*., 2014). Intriguingly, OM‐degrading microorganisms in permanently cold sediments are relatively active at temperatures close to the freezing point (Kostka *et al*., 1999; Arnosti and Jørgensen, 2003; Robador *et al*., 2016), because of psychrophilic adaptations (Knoblauch and Jørgensen, 1999; Cavicchioli, 2016). The microbial communities in Svalbard fjord sediments are highly diverse (Teske *et al*., 2011), and dominated by representatives of the classes *Deltaproteobacteria, Gammaproteobacteria* and the phylum *Bacteroidetes* (Ravenschlag *et al*., 1999, 2000, 2001). Even though a variety of specific members of this arctic sediment microbial community could be isolated (Knoblauch *et al*., 1999; Sahm *et al*., 1999; Wang *et al*., 2015), the identity and population dynamics of most microorganisms that are responsible for the degradation of complex OM under cold, anoxic conditions in marine sediments is unknown.

In this study, we conducted microcosm experiments with ^13^C‐labelled substrates in order to directly link OM degradation processes in arctic marine sediments with specific microorganisms (Supporting Information Fig. S1). We used sediment samples from a fjord in Svalbard (Smeerenburgfjorden, station J) where sulfate reduction is the predominant terminal remineralization process (Finke *et al*., 2007). Anoxic sediment incubations were either supplemented with a low dose (LD) or a high dose (HD) of ^13^C‐labelled cyanobacterial necromass (spirulina) in order to mimic a pulse of bulk OM input to the seabed or ^13^C‐labelled acetate, an important organic carbon degradation intermediate and electron donor for anaerobic respiration in marine sediments. We investigated bacterial community responses to substrate supplementation by amplicon‐sequencing of 16S rRNA genes and transcripts in light of changes in VFA concentrations and sulfate reduction rates, and by single‐cell Raman microspectroscopy. This allowed us to (i) identify species‐level phylotypes involved in the degradation cascade of cyanobacterial necromass and in the terminal oxidation of acetate, (ii) reveal main products of cyanobacterial necromass degradation and provide evidence for their consumption by SRM and microorganisms that use alternative electron acceptors for energy conservation and (iii) confirm the specific incorporation of substrate‐derived ^13^C into individual target cells via a combination of catalyzed reporter deposition fluorescence *in situ* hybridization (CARD‐FISH) and Raman microspectroscopy.

## Results

### *Enhanced sulfate reduction rates in spirulina necromass‐ and acetate‐amended microcosms*

**Figure 1:**
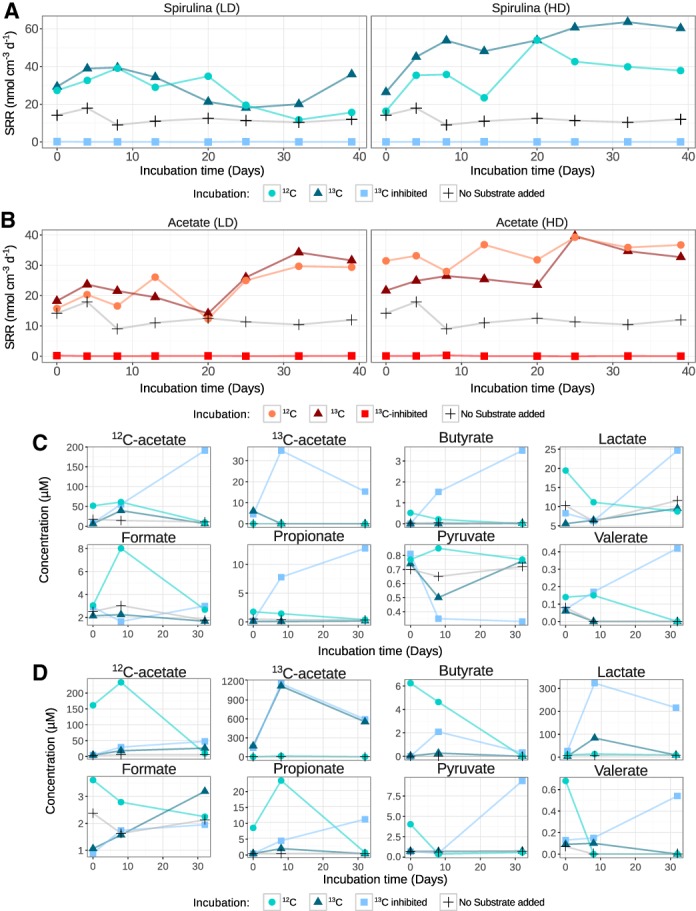
Sulfate reduction rates and volatile fatty acid concentrations in substrate‐supplemented sediment incubations. Sulfate reduction rates (nmol cm^−1^ d^−1^) are shown for incubations supplemented with spirulina (A) and acetate (B). Concentrations of volatile fatty acids are indicated for LD (C) and HD (D) incubations with spirulina. ^13^C‐inhibited, sediment incubations with ^13^C‐substrate and molybdate. LD, low substrate dose (50 µg ml^−1^ spirulina or 50 μM acetate). HD, high substrate dose (1 mg ml^−1^ spirulina or 1 mM acetate). Note that the scales are different for each treatment and VFA.

Sulfate reduction rates in the unamended controls ranged from 9 to 18 nmol cm^−3^ d^−1^ (Fig. 1 A and B). The addition of cyanobacterial necromass significantly enhanced sulfate reduction rates to 18–40 nmol cm^−3^ d^−1^ (*P* < 0.001) and to 45–64 nmol cm^−3^ d^−1^ (*P* < 0.001) in LD and HD ^13^C‐spirulina incubations respectively (Fig. 1A). Amendment of ^13^C‐acetate also significantly increased sulfate reduction rates to 14–34 nmol cm^−3^ d^−1^ (*P* < 0.001) and to 22–40 nmol cm^−3^ d^−1^ (*P* < 0.001) in LD and HD incubations respectively (Fig. 1B). Sulfate reduction rates were below the detection limit in incubations with molybdate as inhibitor (Fig. 1A and B).

### *Acetate is the main volatile fatty acid produced from spirulina degradation*

Average background concentrations of VFAs in the unamended control microcosms were low (12 µM acetate, 9 µM formate, 4 µM lactate and < 1 µM propionate, pyruvate, butyrate and valerate) (Fig. 1 and Supporting Information Fig. S2). The concentrations of VFAs differed substantially between the incubations with ^13^C‐spirulina and ^12^C‐spirulina (Fig. 1C and D), which suggests considerable differences in the composition of the purchased spirulina products (Supporting Information Results and Discussion). Only results from the ^13^C‐spirulina incubations are discussed in the main text. A pulse of ^13^C‐spirulina was added at the beginning of the experiment and its degradation led to the production of mainly ^13^C‐acetate and several other VFAs (Fig. 1C and D). Compared to uninhibited incubations, concentrations of VFAs increased more over time in incubations with the sulfate‐reduction inhibitor molybdate. Exceptions were HD ^13^C‐spirulina incubations where ^13^C‐acetate increased to similarly high concentrations ( > 1.1 mM) with and without molybdate (Fig. 1D). In LD ^13^C‐spirulina incubations, ^13^C‐acetate only accumulated in the presence of molybdate and reached concentrations of 35 µM (Fig. 1C). Other VFAs that consistently accumulated in molybdate‐inhibited ^13^C‐spirulina microcosms were formate, propionate, butyrate and valerate, which reached concentrations of 25 µM, 11 µM, 4 µM and 0.42 µM in LD incubations and 324 µM, 13 µM, 2 µM and 0.52 µM in HD incubations. Pyruvate increased up to 9 µM but only in HD ^13^C‐spirulina incubations (Fig. 1D) and not in LD ^13^C‐spirulina incubations (Fig. 1C). Lactate concentrations were always comparable to the unamended controls (Fig. 1C and D). Intriguingly, low amounts of ^12^C‐acetate accumulated in molybdate‐inhibited ^13^C‐spirulina incubations (up to 191 and 48 µM in LD and HD incubations respectively), indicating that addition of fresh cyanobacterial necromass additionally stimulated the breakdown of endogenous OM (van Nugteren *et al*., 2009).

Concentrations of acetate, formate, propionate, butyrate and valerate remained mostly unchanged in incubations with repeated amendments of acetate (Supporting Information Fig. S2). Similar to spirulina incubations, inhibition with molybdate led to an accumulation of acetate, butyrate, propionate and valerate (Supporting Information Fig. S2). However, the concentrations of these VFAs were consistently lower in molybdate‐inhibited incubations with ^13^C‐acetate than with ^13^C‐spirulina. This further suggests that a substantial part of the VFAs that accumulated in the inhibited ^13^C‐spirulina incubations came from breakdown of the supplemented substrate.

### *An assemblage of eleven phylotypes from diverse taxa is associated with degradation of spirulina necromass under sulfidic conditions*

**Figure 2:**
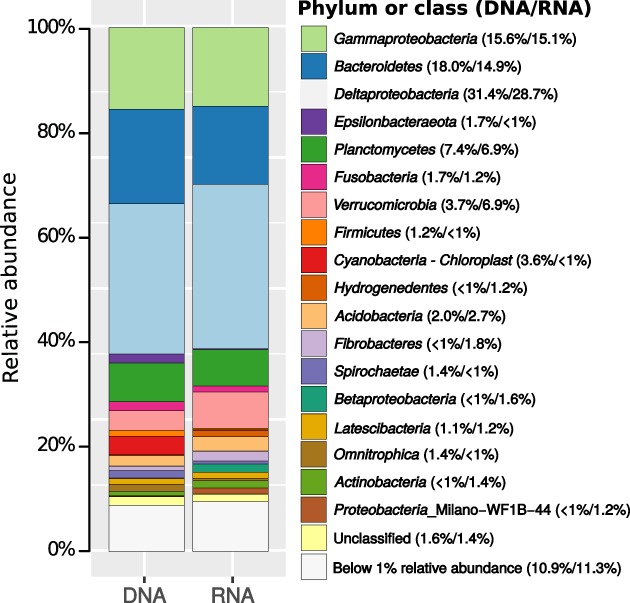
Bacterial community composition in sulfidic Smeerenburgfjorden surface sediment at the start of the incubation experiments (day 0). Average (*n* = 4) relative 16S rRNA gene (DNA) and transcript (RNA) abundance of bacterial phyla or proteobacterial classes. Only phyla/classes with a relative abundance ≥ 1% on DNA or RNA level are indicated.

We monitored changes in the relative abundance and ribosomal activity of bacterial community members during the microcosm experiments by pyrosequencing of 16S rRNA gene and transcript amplicons. The sediment community at the start of the experiment (day 0) was dominated by *Deltaproteobacteria, Bacteroidetes, Gammaproteobacteria* and *Planctomycetes* (Fig. 2); these taxa also harboured 21 of the 25 abundant phylotypes ( ≥ 1% 16S rRNA gene and/or transcript abundance) (Supporting Information Table S1). The other four abundant phylotypes were affiliated with *Fusobacteria, Betaproteobacteria*, candidate group Milano‐WF1B‐44 and *Cyanobacteria‐Chloroplast* (Supporting Information Table S1). Amendments with acetate did not trigger considerable changes in 16S rRNA gene or transcript alpha‐ (Supporting Information Table S2) and beta‐diversity (Supporting Information Fig. S3). In contrast, incubations with ^13^C‐spirulina led to LD‐ and HD‐dependent shifts in 16S rRNA gene and transcript composition (Supporting Information Figs S3 and S4).

**Figure 3:**
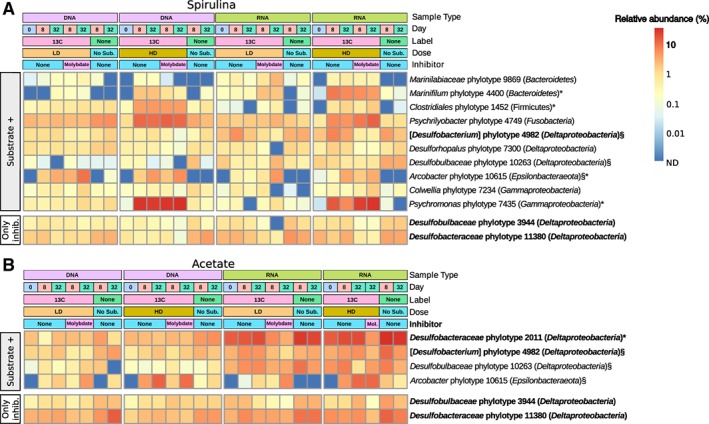
Relative abundance heat‐maps of substrate‐responsive and molybdate‐inhibited 16S rRNA phylotypes. Depicted are phylotypes that responded to substrate amendment and/or molybdate inhibition in spirulina (A) and acetate (B) incubations with significant relative abundance changes in at least two 16S rRNA gene or transcript libraries as compared to unamended or uninhibited incubations. SRM (i.e., molybdate‐inhibited) phylotypes are highlighted in bold. Phylotypes that responded to both spirulina and acetate amendments are indicated with a section sign (§). Phylotypes that were analyzed by CARD‐FISH‐Raman microspectroscopy are indicated with an asterisk (*). Substrate + , phylotypes that responded to substrate additions. Only inhib., phylotypes that did not respond to substrate addition but were inhibited by molybdate. No Sub., no‐substrate control incubation. Mol., Molybdate.

We identified individual phylotypes that responded to the added substrates by comparing their relative 16S rRNA gene and transcript abundances in incubations with substrate versus unamended controls. Overall, eleven responsive phylotypes of the phyla/classes *Gammaproteobacteria, Deltaproteobacteria, Epsilonbacteraeota, Bacteroidetes, Firmicutes* and *Fusobacteria* were identified by two‐proportion *T*‐tests (*P* ≤ 0.01); of which seven were only associated with spirulina amendments, one with acetate amendment alone and three with both substrate amendments (Fig. 3). Ten of these phylotypes had a relative 16S rRNA gene abundance of ≥ 0.1% at the start of the incubations (including four phylotypes with ≥ 1%) (Supporting Information Fig. S5), demonstrating that the majority of the responsive consortium are abundant microbiota members in the sulfidic zone of the arctic marine sediment *in situ.* Five phylotypes (1452, 4400, 4749, 7435, 10615) responded by significant increases in relative abundances of both 16S rRNA genes and transcripts, while six (2011, 4982, 7234, 7300, 9869, 10263) responded only by increases in relative 16S rRNA transcript abundance, including three deltaproteobacterial phylotypes that were stimulated by the amendment of acetate. The fourth, putative acetate‐utilizing *Arcobacter* phylotype 10615 was low in abundance and ribosomal activity ( < 0.1%) at day 0, but became dominant in spirulina‐ and acetate‐amended incubations (Fig. 3). Four deltaproteobacterial phylotypes, including *Desulfobacteraceae* phylotype 2011 and [*Desulfobacterium*] phylotype 4982 that responded to acetate, were considered as putative SRM because they were inhibited by molybdate. The other two SRM phylotypes (*Desulfobacteraceae* phylotype 11380 and *Desulfobulbaceae* phylotype 3944) did not respond significantly to spirulina or acetate amendments (Fig. 3).

### *Single cell stable isotope probing revealed ^13^C‐incorporation in phylotypes that responded to substrate supplementation*

**Figure 4:**
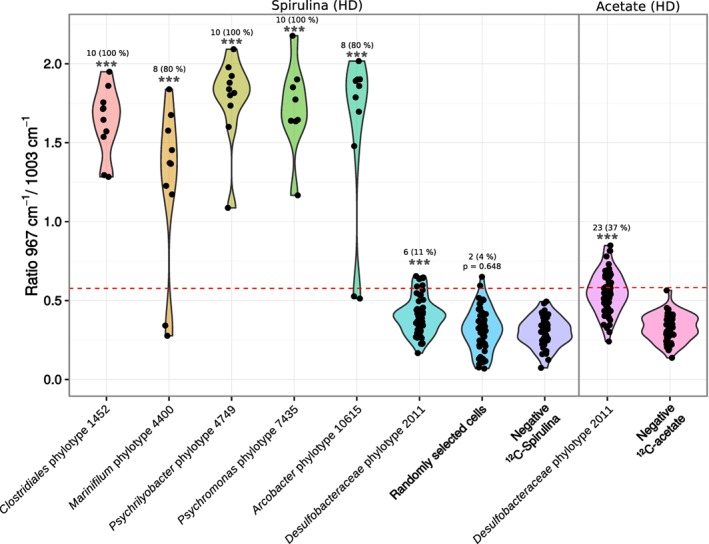
^13^ **C‐labelling of individual cells of substrate responsive phylotypes.** CARD‐FISH‐Raman microspectroscopy analysis of ^13^C‐labelled probe‐targeted cell populations (including selected substrate‐responsive phylotypes) at day 32 in high dose (HD) ^13^C‐spirulina (1 mg ml^−1^) and ^13^C‐acetate (1 mM) incubations. Stars (***) indicate significant (*P* < 0.001) ^13^C‐labelling, as revealed by comparing ^13^C‐labelled target populations to the respective negative control using Wilcoxon Rank Sum tests. Negative ^12^C‐spirulina, randomly selected cells from incubations with ^12^C‐spirulina at day 0. Negative ^12^C‐acetate, cells of *Desulfobacteraceae* phylotype 2011 (*Deltaproteobacteria*) from incubations with ^12^C‐acetate at day 0. The red dashed line shows the threshold for detection of ^13^C‐labelling in an individual cell.

Raman microspectroscopy of randomly selected cells from day 32 of the HD ^13^C‐spirulina incubations showed that only 4% of cells displayed a low level of ^13^C labelling, which supports the finding that only a small part of the native sediment community is involved in the degradation of spirulina necromass (Fig. 4). We further combined CARD‐FISH and Raman microspectroscopy to confirm ^13^C‐substrate utilization and assimilation by individual cells of selected taxa that responded to the substrate additions. The used or newly developed CARD‐FISH probes covered four putative hydrolytic/fermenting phylotypes, i.e., *Clostridiales* phylotype 1452, *Marinifilum* phylotype 4400, *Psychrilyobacter* phylotype 4749 and *Psychromonas* phylotype 7435 and two putative acetate‐utilizing phylotypes, i.e., *Desulfobacteraceae* phylotype 2011 and *Arcobacter* phylotype 10615 (Supporting Information Fig. S6). All phylotypes that responded to ^13^C‐spirulina, including *Arcobacter* phylotype 10615 that responded to ^13^C‐spirulina and ^13^C‐acetate, showed significant cellular ^13^C‐incorporation at day 32 of the HD ^13^C‐spirulina incubations (Fig. 4). *Desulfobacteraceae* phylotype 2011 was also significantly enriched in comparison to the ^12^C‐spirulina control but only a small fraction (11%) of the cells were labelled at low level (Fig. 4). The fraction of ^13^C‐labeled *Desulfobacteraceae* phylotype 2011 cells was larger (37%) in HD ^13^C‐acetate incubations but individual cells had low ^13^C/^12^C phenylalanine peak height ratios, indicating only minor ^13^C‐incorporation.

## Discussion

### *Different cellular responses of members of a bacterial consortium involved in mineralization of organic matter in an arctic marine sediment*

OM in anoxic, sulfidic marine sediments is mainly degraded by the joint activity of hydrolysers, fermenters and SRM (Arndt *et al*., 2013). In order to investigate the microorganisms involved in sequential OM degradation, we performed anoxic incubation experiments at 0°C with arctic marine sediments from Smeerenburgfjorden (Svalbard). In these sediments sulfate reduction is the dominant carbon mineralization process, with important contributions by iron reduction (Finke *et al*., 2007). Sulfate reduction rates in sediment incubations without external substrates (9–18 nmol cm^−3^ d^−1^) (Fig. 1A and B) were in the range of previously determined mean *in situ* rates of 15–20 nmol cm^−3^ d^−1^ in the sulfate reduction zone of Smeerenburgfjorden sediments (Jørgensen *et al*., 2010; Sawicka *et al*., 2012; Robador *et al*., 2016). Supplementation of spirulina and acetate, each at two different doses, to individual sediment microcosms immediately triggered significant increases in sulfate reduction rates, which were more pronounced in spirulina incubations (Fig. 1A). Interestingly, a 20‐fold increase in substrate concentration resulted in only 55% and 38% increased sulfate reduction rates in spirulina and acetate incubations respectively. This indicated that the SRM community had almost reached maximal physiological activity at the lower substrate dose. Hydrolysis and fermentation of spirulina biomass predominantly generated acetate and smaller amounts of butyrate, propionate, valerate, formate and pyruvate (Fig. 1C and D). Accumulation of these VFAs in sulfate reduction‐inhibited incubations confirmed that mineralization of OM and utilization of VFAs in this sediment is largely dependent on the activity of SRM.

Smeerenburgfjorden sediments harbor a cold‐adapted microbial community that is primarily composed of *Deltaproteobacteria, Gammaproteobacteria* and representatives of the phylum *Bacteroidetes* (Ravenschlag *et al*., 1999, 2000, 2001). These classes/phyla were also dominant in the sediments analyzed in the present study (Fig. 2). Here, we show that only eleven species‐level phylotypes of this complex sediment microbiota responded significantly to spirulina or acetate supplementation during a 38 days incubation period at 0°C. Responsive phylotypes either only increased in relative 16S rRNA transcript abundance, which suggests response by increased ribosomal activity, or additionally in relative 16S rRNA gene abundance, which suggests response by growth (Hausmann *et al*., 2016). Acetate mainly triggered increases in ribosomal activity in SRM phylotypes 2011 and 4982, whereas four (phylotypes 4400, 1452, 4749, 7435) of the six phylotypes involved in the fermentation of spirulina necromass and the acetate‐utilizing, non‐sulfate‐reducing *Arcobacter* phylotype 10615, also responded by increased relative 16S rRNA gene abundance (Fig. 3). Differences in spirulina‐ and acetate‐based ^13^C‐labeling of individual cells confirmed different cellular response patterns of the diverse functional guilds in anaerobic carbon decomposition (Fig. 4). This difference in cellular response could relate to frequently observed higher process rates of hydrolysis as compared to the mineralization of VFAs (Arnosti, 2011). Hydrolytic microorganisms in marine sediments can respond to increased substrate availability by synthesis of extracellular enzymes (Brüchert and Arnosti, 2003), which might even persist in sediments and enable a fast response to new OM input (Fabiano and Danovaro, 1998). It has also been speculated that glucose‐utilizing fermenters in arctic marine sediments have substantially higher growth yields than acetate‐utilizing SRM (Kirchman *et al*., 2014). SRM in marine sediments require several days to upregulate their VFA turnover (Arnosti *et al*., 2005). The calculated energy yield from acetoclastic sulfate reduction at *in situ* conditions in Smeerenburgfjorden sediment (Supporting Information Material and Methods; Table S3) was −93.3 kJ mol^−1^, which is much higher than the energetic limit of this process of −30 kJ mol^−1^ (Jin and Bethke, 2009; Glombitza *et al*., 2015). Thus, SRM in our sediment microcosms seemed not to be energy‐limited even at low *in situ* acetate concentrations. However, most of the substrate might be funnelled through catabolic pathways for energy conservation as opposed to also supplying elements and energy for anabolism and thus growth and cell division (Rabus *et al*., 2013). Additionally, doubling times of previously isolated, psychrophilic SRM at 0°C are considerably lower than at their optimal growth temperatures, i.e., 30–170 h at 7–18°C (Knoblauch and Jørgensen, 1999). Cell physiological constraints of SRM might further limit the uptake of substrate from the sediment and intracellular activation (Schauder *et al*., 1986; Glombitza *et al*., 2015), and also adsorption of VFAs by the sediment matrix can significantly reduce substrate uptake and mineralization (Shaw *et al*., 1984; Finke *et al*., 2007). Increase in relative 16S rRNA gene abundance, i.e., putative growth of the non‐sulfate‐reducing *Arcobacter* phylotype in acetate incubations (Fig. 3) is conflicting. However, this may be explained by the use of the energetically more favourable electron acceptors manganese, nitrate or iron, which result in higher energy yields during OM oxidation than the use of sulfate (Froelich *et al*., 1979; LaRowe and Amend, 2014).

### *Psychrophilic fermenters of cyanobacterial necromass*

**Figure 5:**
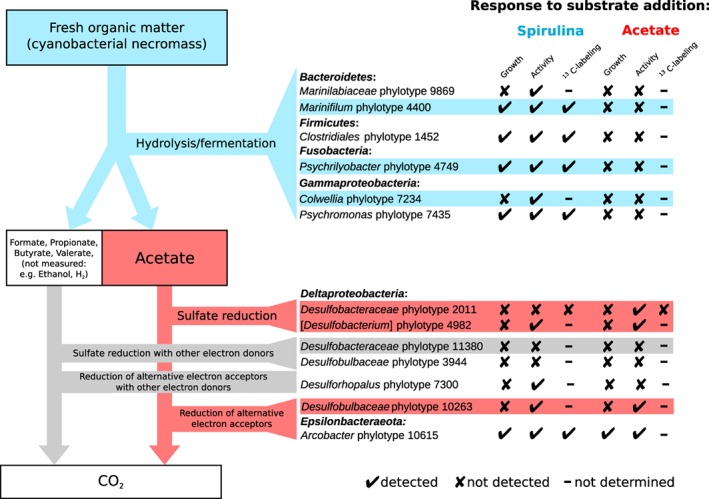
A bacterial interaction model for organic matter degradation in sulfidic arctic marine sediments of Smeerenburgfjorden, Svalbard. Fresh biomass that is introduced into the system by sedimentation is utilized by hydrolytic and fermentative bacteria and degraded to VFAs, mainly acetate, which are further utilized by SRM or microorganisms that use alternative electron acceptors. Putative roles in organic carbon degradation are shown for identified phylotypes (Fig. 3). Growth, significant response in relative 16S rRNA gene abundance. Activity, significant response in relative 16S rRNA transcript abundance. ^13^C‐labelling, significant ^13^C‐incorporation by > 50% of phylotype cells from ^13^C‐spirulina or ^13^C‐acetate as revealed by single‐cell CARD‐FISH‐Raman (Fig. 4). Responsive phylotypes that are highly abundant and/or ribosomally active in native sediment, i.e., ≥ 1% abundance in 16S rRNA gene or transcript libraries at day 0 (Supporting Information Table S1), are shaded.

Six phylotypes were considered hydrolysers/fermenters, because they showed a significant response to spirulina amendments, did not respond to the amendment of acetate, were not negatively impacted by amendment of the sulfate reduction inhibitor molybdate, and were related to described species with hydrolytic and/or fermentative metabolic capabilities (Fig. 5). Four of the phylotypes (*Psychromonas* phylotype 7435, *Gammaproteobacteria*; *Psychrilyobacter* phylotype 4749, *Fusobacteria*; *Marinifilum* phylotype 4400, *Bacteroidetes*; *Clostridiales* phylotype 1452, *Firmicutes*) grew (Fig. 3A) and incorporated spirulina‐derived ^13^C into their cells (Fig. 4). These phylotypes thus seemed to be primarily r‐selected (Lynch and Neufeld, 2015) and thus able to quickly take advantage and grow upon availability of larger amounts of OM that, for example, are available during spring bloom‐like events of primary producers that deposit to the seafloor upon cell death (Hebbeln and Wefer, 1991). In contrast, *Colwellia* phylotype 7234 (*Gammaproteobacteria*) and *Marinilabiliaceae* phylotype 9869 (*Bacteroidetes*) only responded by relative increase in ribosomal activity (Fig. 5). *Colwellia* phylotype 7234 was of high relative abundance in the native sediment (day 0) (Supporting Information Table S1), which suggests that it retains a more stable population size in periods of low nutrient supply.

Bacterial cellular biomass is composed of thousands of individual molecules and metabolites (Engelking, 2015), but amendment of spirulina necromass activated only very few members of the complex sediment microbiota, as observed in another study (Graue *et al*., 2012). Dry spirulina cell extract is mostly composed of crude protein (64‐74%), carbohydrates (12‐20%) and lipids (9‐14%) (Ciferri, 1983), which thus represent the dominant food sources for the responsive bacteria and is in agreement with the various fermentative capabilities of their phylogenetically most closely related species (Supporting Information Results and Discussion).

### *SRM utilizing acetate and other electron donors*

All four phylotypes that were inhibited by molybdate, and thus identified as SRM (Fig. 5), are members of the class *Deltaproteobacteria* (Fig. 3B), which contains the majority of ecologically relevant SRM in marine sediments (Wasmund *et al*., 2017). The abundant *Desulfobacteraceae* phylotype 2011 belongs to the globally distributed Sva0081 group of yet uncultured sediment bacteria (Supporting Information Fig. S5). Phylotype 4982 (*Desulfobulbaceae*) is very closely related to [*Desulfobacterium*] *catecholicum* (99% 16S rRNA identity) (Supporting Information Fig. S5), a complete oxidizer of acetate and other VFAs (Szewzyk and Pfennig, 1987). Both phylotypes responded to acetate amendment but mainly by increased ribosomal activity (Fig. 3B). Additionally, *Desulfobacteraceae* phylotype 2011 incorporated only small amounts of ^13^C from acetate into its biomass (Fig. 4). Our results suggest that cellular activity of acetate‐utilizing SRM in arctic marine sediments is largely decoupled from an increase in their population size. We postulate that populations of SRM in marine surface sediments with constantly cold temperatures are rather stable (Robador *et al*., 2009), and only change slowly in response to long‐term changes in substrate availability.

Besides the two acetate‐utilizing SRM‐phylotypes, we also identified *Desulfobulbaceae* phylotype 3944 and *Desulfobacteraceae* phylotype 11380 to be suppressed in incubations with molybdate (Fig. 3). Both phylotypes did not respond significantly to either spirulina or acetate amendments, which suggests that they utilize other, endogenous electron donors for sulfate reduction (Fig. 5). Alternatively, they might have other energy metabolisms that are inhibited by molybdate, e.g., utilization of taurine (Lie *et al*., 1999) or disproportionation of thiosulfate or elemental sulfur (Finster *et al*., 1998).

### *Bacteria that utilize VFAs but are not strictly dependent on sulfate reduction*

Three VFA‐utilizing phylotypes were not suppressed by molybdate (Fig. 5); *Arcobacter* phylotype 10615, *Desulfobulbaceae* phylotype 10263 and *Desulforhopalus* phylotype 7300. The first two responded to spirulina and acetate amendments (Figs 3 and 4), which indicated that both phylotypes primarily utilized the spirulina fermentation product acetate. In contrast *Desulforhopalus* phylotype 7300 only responded to spirulina incubations (Fig. 3A), potentially by consuming spirulina degradation intermediates other than acetate. Given that *Arcobacter* phylotype 10615 responded to acetate with particularly massive relative abundance increases (Fig. 3B), we hypothesize that it used electron acceptors more energetically favorable than sulfate for the oxidation of VFAs (Fig. 5). *Arcobacter*‐related microorganisms use manganese, iron or nitrate as electron acceptors (Thamdrup *et al*., 2000; Vandieken *et al*., 2012; Canion *et al*., 2013; Vandieken and Thamdrup, 2013). Also, the SRM *Desulfobulbus propionicus*, which is related to *Desulfobulbaceae* phylotype 10263 (94.4% 16S rRNA identity) (Supporting Information Fig. S5), has similar metabolic capabilities in the absence of sulfate (Dannenberg *et al*., 1992; Lovley and Phillips, 1994; Holmes *et al*., 2004). *Desulforhopalus* phylotype 7300 was closely related to *Desulforhopalus singaporensis* (Supporting Information Fig. S5), which besides sulfate can also use nitrate, sulfite and thiosulfate as electron acceptor (Lie *et al*., 1999). Iron and nitrate reduction contribute to organic carbon oxidation in Svalbard fjord sediments (Kostka *et al*., 1999; Finke *et al*., 2007; Canion *et al*., 2014) and many SRM use intermediate sulfur species for respiration (Wasmund *et al*., 2017). Thus, iron, nitrate or intermediate sulfur species might have been utilized by the phylotypes that were not inhibited by molybdate.

## Conclusions

We investigated degradation of complex OM in sulfidic Smeerenburgfjorden sediment from the arctic archipelago of Svalbard. We propose a metabolic and species interaction model for dead biomass mineralization in the conditions studied that may be found in permanently cold and sulfate‐reduction‐dominated sediments (Fig. 5). Deposited organic biomass was primarily degraded by a limited number of species from diverse phyla. Initial hydrolysis and fermentation of mainly proteins, carbohydrates and lipids to diverse VFAs were catalyzed by *Psychrilyobacter* (*Fusobacteria*), *Marinifilum* and *Marinilabiaceae* (*Bacteroidetes*), *Colwellia* and *Psychromonas* (*Gammaproteobacteria*) and *Clostridiales* species (*Firmicutes*). Acetate, the main fermentation product, was mineralized by sulfate‐reducing species of the families *Desulfobacteraceae* and *Desulfobulbaceae* (*Deltaproteobacteria*) and by *Arcobacter* (*Epsilonbacteraeota*) and *Desulfobulbaceae (Deltaproteobacteria)* species that presumably used other electron acceptors than sulfate. Interestingly, cellular activation (i.e., increases in relative 16S rRNA transcript abundance and in single‐cell ^13^C‐labelling) upon availability of new substrate was coupled to relative population growth (i.e., increases in relative 16S rRNA gene abundance) in microorganisms able to directly hydrolyze/ferment cyanobacterial necromass and in the acetate‐utilizing *Arcobacter* species, but not in acetate‐utilizing SRM. These different cellular responses of each species have important implications for the population dynamics of the different functional guild members in marine sediments. It is tempting to speculate that the taxa identified here will play increasing roles in the future mineralization of the predicted increased phyto‐detrital organic material in arctic benthic systems (Lalande *et al*., 2009; Sørensen *et al*., 2015), and in determining the extent of burial of organic material into the subsurface.

## Experimental procedures

### *Marine sediment sampling*

Arctic marine sediment samples were collected from Smeerenburgfjorden, Svalbard (station J; 79°43′N, 11°05′E; water depth 216 m) in August 2011. Sediment from 5 to 10 cm depth was collected from several HAPS cores (KC Denmark A/S, Silkeborg, Denmark) (Kanneworff and Nicolaisen, 1972), transferred into gas‐tight plastic bags (Hansen *et al*., 2000) and stored on ice at 0°C for up to 2.5 months prior to incubations.

### *Sediment incubations*

Approximately 2 l of anoxic sediment slurry was prepared per set of substrate and concentrations (Supporting Information Fig. S1A) by homogenizing sediment with anoxic bottom water from station J at a 1:1 (w/w) ratio under constant flow of N_2_ gas. 200 ml of slurry was distributed into 250 ml serum bottles, amended with different substrates, sealed with butyl rubber stoppers (Geo‐Microbial Technologies) and incubated at 0°C (Supporting Information Fig. S1A). Incubations were performed with either acetate or spirulina (Sigma‐Aldrich, Steinheim, Germany). Spirulina consists of freeze‐dried cells of the cyanobacterium *Arthrospira platensis.* Incubations were performed with two different substrate concentrations; i.e., a low dose (LD) and a high dose (HD) with 50 µg ml^−1^ and 1 mg ml^−1^ of spirulina and with 50 µM and 1 mM of acetate respectively (Supporting Information Fig. S1A). Spirulina was added as a one‐time pulse at the beginning of the experiment, whereas acetate was continuously added every four to seven days (Supporting Information Fig. S1B) in order to compensate for the supposedly high acetate turnover (Finke *et al*., 2007). LD acetate supplementation was in the range of concentrations measured *in situ* in Svalbard sediments (Finke *et al*., 2007). Parallel incubations were performed with ^13^C‐labeled acetate or ^13^C‐labelled spirulina (both Sigma Aldrich, 99 atom% ^13^C), ^12^C‐substrates (unlabeled control), ^13^C‐labelled substrate in combination with 5 mM molybdate as a sulfate‐reduction inhibitor (inhibited control) and an unamended control incubation (no‐substrate control) (Supporting Information Fig. S1A). Molybdate was added at day 0 and after 3 weeks of incubation. At days 0, 4, 8, 13, 20, 25, 32 and 39 after the start of the incubations, subsamples were taken through the rubber stopper with a sterile 16‐gauge needle (Braun) attached to a 10 ml Omnifix syringe (Braun). At each sampling time point, a subsample of 6.5 ml was taken and stored either at −80°C for VFA and nucleic acid analyses or fixed for CARD‐FISH analyses as described below.

### *Biogeochemical analyses*

Concentrations of the VFAs acetate, formate, propionate, butyrate, valerate, lactate and pyruvate were measured in the supernatant by 2‐dimensional ion chromatography‐mass spectrometry (IC‐IC‐MS; Dionex ICS‐3000 coupled to an MSQ Plus™, both Thermo Scientific), equipped with an Ion Pack™ AS 24 as the first column to separate the VFAs from chloride, and an Ion Pack™ AS 11 HC as the second column) (Glombitza *et al*., 2014). Prior to the analyses, samples were thawed and filtered through disposable syringe filters (Acrodisc™ 13 mm, IC grade, polyethersulfone (PES) membrane with 0.2 µM pore size), that were previously cleaned by rinsing with 10 ml Milli‐Q water (Utrapure, Type 1) (Glombitza *et al*., 2014). Both isotopic variants of acetate were analyzed in parallel in separate selected ion monitoring (SIM) channels, i.e., ^12^C‐acetate (m/z 59) and ^13^C‐acetate (m/z 61). The Gibbs Energy of acetoclastic sulfate reduction was calculated as described in Supporting Information Materials and Methods. Parallel incubations were set up with ^35^S‐labelled carrier‐free sulfate tracer and sulfate reduction rates were determined using a single‐step cold chromium distillation method (Kallmeyer *et al*., 2004). Significance of SRR differences between no substrate controls and substrate amended incubations were determined by Wilcoxon tests using the function wilcox.test() from the R statistical package (R Core Team, 2015).

### *16S rRNA gene and transcript sequence analyses*

Nucleic acid extraction and preparation of 16S rRNA gene and transcript libraries is described in Supporting Information Material and Methods. Sequencing was performed on a GS FLX + instrument using Titanium chemistry (Roche, Mannheim, Germany) by Eurofins MWG Operon (Ebersberg, Germany). Sequencing reads were filtered using mothur's implementation of PyroNoise (Schloss *et al*., 2009; Quince *et al*., 2011) and clustered into phylotypes of approximate species‐level at 97% sequence identity using UCLUST (Edgar, 2010) as evaluated previously (Berry *et al*., 2012). Representative sequences (mean length of 220 bp) were aligned with mothur using default settings. Chimeras that were detected with Chimera Slayer (Haas *et al*., 2011) were excluded from further analysis. Altogether we obtained 344,867 high quality reads (on average 4732 reads per sample) that clustered into 11,548 species‐level phylotypes. Phylum/class‐level classification was performed using mothur's implementation of the Ribosomal Database Project naïve Bayesian classifier (Wang *et al*., 2007; Schloss *et al*., 2009) with default settings. Note that reclassification of the class *Epsilonproteobacteria* into the new phylum *Epsilonbacteraeota* was recently proposed (Waite *et al*., 2017). Alpha diversity metrics were calculated (Caporaso *et al*., 2010) with re‐sampling (100 re‐samples) at 3250 reads to avoid sample based artifacts (Lozupone *et al*., 2011). Principal coordinates analysis (PCoA) was performed in the R software environment (R Core Team, 2015) on the re‐sampled phylotype table based on Bray‐Curtis dissimilarities using the software package phyloseq (McMurdie and Holmes, 2013). Phylotypes with a relative abundance of ≥ 1% of the community in at least one sample were aligned against the SILVA database SSU_Ref_NR99_128 (Quast *et al*., 2013) using SINA (Pruesse *et al*., 2012). A phylogenetic tree was calculated with FastTree (Price *et al*., 2009) using an alignment of closely related sequences that were selected from the non‐redundant SILVA database SSU_Ref_NR99_128 (Quast *et al*., 2013). Phylotype sequences were subsequently added to the resulting tree using the EPA algorithm (Berger *et al*., 2011) in RAxML (Stamatakis, 2014). Trees were visualized using iTOL (Letunic and Bork, 2016). The sequence similarity between phylotypes and representative strains in the non‐redundant SILVA database SSU_Ref_NR99_128 (Quast *et al*., 2013) was calculated using t_coffe (Notredame *et al*., 2000). Phylotypes associated with the utilization of the added substrate at a given dose or with sulfate reduction had to be significantly enriched in at least two 16S rRNA gene and/or transcript libraries (*n* = 4 for substrate utilization, *n* = 8 for sulfate reduction) compared to the respective control (no substrate control or inhibited control). Significant enrichment of phylotypes was determined using a two‐proportion *T*‐test (Müller *et al*., 2014; Robador *et al*., 2016). *P*‐values were corrected for multiple comparisons using the Benjamini‐Hochberg false discovery rate method with p.adjust() from the R statistical package (R Core Team, 2015). Corrected P‐values of ≤ 0.01 were considered significant.

### *Sequence data*

16S rRNA gene and transcript sequence data is available in the NCBI Short Read Archive under accession number SRP133170.

### *Catalyzed reporter deposition‐fluorescence in situ hybridization coupled with raman microspectroscopy (CARD‐FISH‐raman)*

Microbial cells were extracted from arctic marine sediment as described in Supporting Information Materials and Methods. The extracted cells were collected by filtration on polycarbonate filters (GTTP type, pore size 0.2 µm, Millipore) and identified by CARD‐FISH or two‐step CARD‐FISH (Supporting Information Materials and Methods). Hybridized cells were extracted from the filters (Supporting Information Materials and Methods) and spotted onto an aluminum‐coated slide (A1136; EMF Corporation) for analysis on a LabRAM HR800 confocal Raman microscope (Horiba Jobin‐Yvon) as outlined previously (Huang *et al*., 2007). The obtained single cell spectra were baseline‐corrected using a polynomial function. Spectra were then aligned according to the phenylalanine peak region and normalized by dividing the spectral intensity at each wavelength by the total spectral intensity. Ratios between the height of the ^13^C‐phenylalanine peak (wavelength of 960–970 cm^−1^) and the height of the ^12^C‐phenylalanine peak (wavelength of 1000–1005 cm^−1^) were calculated. Significance of the peak height ratio differences between ^13^C‐labeled probe‐targeted cell populations and negative controls were determined in the R software environment (R Core Team, 2015) with wilcoxon.test(). Testing for spirulina uptake was performed between probe‐targeted cells in ^13^C‐spirulina incubations and populations of cells randomly selected from ^12^C‐spirulina incubations. Testing for acetate uptake by *Desulfobacteraceae* phylotype 2011 was performed between populations from ^13^C‐acetate and ^12^C‐acetate incubations respectively. The threshold for detection of ^13^C‐labeled cells was calculated by adding three times the standard deviation of the negative (^12^C) control to the mean of the negative control.

## Supporting information


**Fig. S1.** Experimental overview.
A. Setup of anoxic sediment incubations.
B. Timeline for substrate additions and sampling. LD, low substrate dose (50 μg ml^‐1^ spirulina and 50 μM acetate). HD, high substrate dose (1 mg ml^‐1^ spirulina and 1 mM acetate). SR‐inhibitor, the sulfate reduction inhibitor molybdate.
**Fig. S2.** Volatile fatty acids concentrations in acetate incubations. Concentrations of volatile fatty acids in (A) LD and (B) HD incubations with acetate. ^13^C‐inhibited, sediment incubations with ^13^C‐substrate and molybdate. Note that acetate was periodically added (black arrows) right before the measurement. LD, low dose (50 μM) of acetate. HD, high dose (1 mM) of acetate. Note that the scales are different for each treatment and each VFA.
**Fig. S3.** Principal coordinates analysis of bacterial beta‐diversity in anoxic sediment incubations. The analyses were based on Bray‐Curtis dissimilarities of relative 16S rRNA gene (DNA) and transcript (RNA) abundances of bacterial phylotypes. Sample colour indicates type and concentration of added substrate. Sample shape indicates the day of sampling.
**Fig. S4.** Bacterial community dynamics in spirulina and acetate amended anoxic sediment incubations. Only phyla/classes with a relative 16S rRNA gene (DNA) and/or transcript (RNA) abundance of ≥ 1% at day 0 are indicated. ^13^C., sediment incubations with ^13^C‐substrate. ^13^Ci., sediment incubations with ^13^C‐substrate and molybdate. ^12^C., sediment incubations with ^12^C‐substrate. None., no‐substrate control. LD, low substrate dose (50 μg ml^−1^ spirulina or 50 μm acetate). HD, high substrate dose (1 mg ml^−1^ spirulina or 1 mM acetate).
**Fig. S5.** Phylogeny of abundant phylotypes. Only phylotypes with ≥ 1% relative 16S rRNA gene or transcript abundance in at least one incubation sample are shown. The tree was calculated using FastTree (Price *et al*., 2009) and an alignment of close relatives of phylotypes selected from the SILVA database SSU_Ref_NR99_128 (Quast *et al*., 2013). Short amplicon sequences were subsequently aligned with SINA (Pruesse *et al*., 2012) and added to the reference tree without changing its topology using the EPA algorithm (Berger et al., 2011) in RAxML (Stamatakis, 2014). Numbers in parentheses indicate average relative 16S rRNA gene/transcript abundances at day 0. Blue and red squares indicate significant enrichment (*P* ≤ 0.01) in spirulina and acetate incubations respectively, compared to the no substrate control. Numbers within the squares give the number of samples (of 24 spirulina and 22 acetate incubation samples in total) in which the phylotype was significantly enriched. Yellow squares indicate sulfate‐reduction‐associated phylotypes, numbers in the square indicate numbers of samples (of 15 in total) in which the phylotype was significantly enriched in uninhibited incubations with substrate compared to molybdate‐inhibited controls.
**Fig. S6.** Raman spectra of responsive phylotypes and their relative sequence abundance.
A. Overlays of single cell Raman spectra are displayed for the CARD‐FISH‐ probe‐labelled phylotypes in ^13^C‐spirulina (1 mg ml^−1^) or ^13^C‐acetate (1 mM) incubations. *Clostridiales* phylotype 1452 (*Firmicutes), Marinifilum* phylotype 4400 (*Bacteroidetes), Psychrilyobacter* phylotype 4749 (*Fusobacteria), Psychromonas* phylotype 7435 (*Gammaproteobacteria), Arcobacter* phylotype 10615 (*Epsilonbacteraeota*) and *Desulfobacteraceae* phylotype 2011 (*Deltaproteobacteria*). Random, Raman spectra from randomly selected cells in the ^13^C‐spirulina (1 mg ml^−1^) incubations. Negative ^13^C‐spirulina, Raman spectra from randomly selected cells from the ^12^C‐spirulina incubations. Negative ^13^C‐ acetate, Raman spectra from cells of *Desulfobacteraceae* phylotype 2011 (*Deltaproteobacteria*) from incubations with ^12^C‐acetate at day 0. Insets show enlarged Raman spectrum regions containing ^13^C‐phenylalanine (wavelength of 960–970 cm^−1^) and ^12^C‐phenylalanine (wavelength of 1000–1005 cm^−1^) peaks.
B. Relative 16S rRNA gene and transcript abundance of CARD‐FISH targeted cell populations in the respective substrate supplemented sediment incubation.
**Table S1.** Relative abundances of phylotypes with ≥ 1% of all bacterial 16S rRNA genes or transcripts at day 0.
**Table S2.** Read number, coverage and alpha‐diversity of bacterial 16S rRNA gene and transcript libraries.
**Table S3.** Data for calculation of *in situ* Gibbs energy of acetoclastic sulfate reduction.
**Table S4.** Thermodynamic properties used for the calculation of standard state Gibbs energy change of reaction [DG_**0**_ (*T, p*)]. Using the SUPCRT92 software (Johnson *et al*., 1992). References: (a) Schock, (1995) and (b) Schock and Helgeson (1988).
**Table S5.** 16S rRNA‐targeted FISH probes.
