# Supplementary material for: Bacterial interactions during sequential degradation of cyanobacterial necromass in a sulfidic arctic marine sediment
Source: Environ Microbiol. 2018 Sep 3;20(8):2927–40. doi: 10.1111/1462-2920.14297 (PMC6175234; doi:10.1111/1462-2920.14297)
Supplement: Supplementary file 1 — Fig. S1. Experimental overview. A. Setup of anoxic sediment incubations. B. Timeline for substrate additions and sampling. LD, low substrate dose (50 μg ml‐1 spirulina and 50 μM acetate). HD, high substrate dose (1 mg ml‐1 spirulina and 1 mM acetate). SR‐inhibitor, the sulfate reduction inhibitor molybdate. Fig. S2. Volatile fatty acids concentrations in acetate incubations. Concentrations of volatile fatty acids in (A) LD and (B) HD incubations with acetate. 13C‐inhibited, sediment incubations with 13C‐substrate and molybdate. Note that acetate was periodically added (black arrows) right before the measurement. LD, low dose (50 μM) of acetate. HD, high dose (1 mM) of acetate. Note that the scales are different for each treatment and each VFA. Fig. S3. Principal coordinates analysis of bacterial beta‐diversity in anoxic sediment incubations. The analyses were based on Bray‐Curtis dissimilarities of relative 16S rRNA gene (DNA) and transcript (RNA) abundances of bacterial phylotypes. Sample colour indicates type and concentration of added substrate. Sample shape indicates the day of sampling. Fig. S4. Bacterial community dynamics in spirulina and acetate amended anoxic sediment incubations. Only phyla/classes with a relative 16S rRNA gene (DNA) and/or transcript (RNA) abundance of ≥ 1% at day 0 are indicated. 13C., sediment incubations with 13C‐substrate. 13Ci., sediment incubations with 13C‐substrate and molybdate. 12C., sediment incubations with 12C‐substrate. None., no‐substrate control. LD, low substrate dose (50 μg ml−1 spirulina or 50 μm acetate). HD, high substrate dose (1 mg ml−1 spirulina or 1 mM acetate). Fig. S5. Phylogeny of abundant phylotypes. Only phylotypes with ≥ 1% relative 16S rRNA gene or transcript abundance in at least one incubation sample are shown. The tree was calculated using FastTree (Price et al., 2009) and an alignment of close relatives of phylotypes selected from the SILVA database SSU_Ref_NR99_128 (Quast et al., 2013). Short amplicon [file EMI-20-2927-s001.pdf]

Supplementary Information

Bacterial interactions during sequential degradation of cyanobacterial necromass in a sulfidic arctic marine sediment

Albert L. Müller, Claus Pelikan, Julia R. de Rezende, Kenneth Wasmund, Martina Putz, Clemens Glombitza, Kasper U. Kjeldsen, Bo B. Jørgensen, and Alexander Loy

Supplementary Materials and Methods

Gibbs Energy calculations

The Gibbs energy (ΔG_r) of acetoclastic sulfate reduction (Eq 1) was calculated according to equation (2):

$$CH_3COO^- + SO_4^{2-} \rightarrow 2\,HCO_3{}^- + HS^- \qquad (1)$$

$$\Delta G_r = \Delta G_r^0 + RT \ln \left(\frac{\prod_i a_{(product)}{}^{\nu_i}}{\prod_i a_{(educt)}{}^{\nu_i}} \right) \qquad (2)$$

In Eq. (2), *R* (0.008314 kJ mol^{-1} K^{-1}) is the universal gas constant, *T* (in K) is the temperature, *a* denotes the activities of the reaction participants (educts and products), and *v* is the stoichiometric coefficient of the *i*-th educt or product. The activities were approximated by multiplying the measured concentrations of the species by their activity coefficients. Activity coefficients were calculated from an extended version of the Debye-Hückel equation (Helgeson, 1969) using the Geochemists Workbench® Software (www.gwb.com) for an ionic strength of 0.7 (S=34) and a temperature of 2°C (275.15 K) estimated from previously published *in situ* temperatures (i.e. 0.67 for bicarbonate and acetate, 0.17 for sulfate and

0.63 for sulfide). The bottom water temperatures at this location was reported to be 3.3°C (Wehrmann *et al.*, 2017), and temperature in the sediment to be 0.4°C (Supplementary Table S3). Chemical species concentrations (except for acetate reported in this study) were taken from previously published pore water data (sulfate: 26.5 mM; DIC: 3 mM; sulfide: 2 µM) (Supplementary Table S3). The standard Gibbs energy of reaction, ΔG_r^0, is a function of temperature (T) and pressure (p). Temperature was taken from the previous published measurements; pressure was approximated by 1 bar. Values of ΔG_r^0 (T, p) were calculated using the revised-HKF (Helgeson-Kirkham-Flowers model) equations of state (Helgeson *et al.*, 1981; Tanger and Helgeson, 1988; Shock and Helgeson, 1990) and the software package SUPCRT92 (Johnson *et al.*, 1992) with thermodynamic data taken from Shock and Helgeson (1988) and Shock (1995) (Supplementary Table S4).

Nucleic acid extraction and preparation of 16S rRNA gene and transcript amplicon libraries

DNA was extracted with the PowerSoil®DNA isolation kit (MO BIO Laboratories, Inc., Carlsbad, CA, USA). RNA from 500 µl of sediment slurry was extracted using an established protocol (Lueders *et al.*, 2004) and resuspended in 100 µl RNase free H_2O. Co-extracted DNA was digested with the TURBO DNA-*free*™ kit (Ambion, Austin, TX, USA) and RNA was re-extracted with TRIzol (Invitrogen, Darmstadt, Germany) according to the manufacturer's instructions. PCR was performed using the primer pair 909F/1492R that target the V6–V9 region of most bacteria using a two-step PCR barcoding approach (Berry *et al.*, 2011, 2012) with low cycle numbers (20+5 cycles) to minimize PCR bias. For the RNA extractions, the Access RT-PCR System (Promega, Madison, WI, USA) was used instead of the first PCR step according to the manufacturer's instructions (but with only 20 PCR cycles). 16S rRNA gene and transcript amplicon libraries were constructed from each incubation at days 0, 8, and 32. PCR products were purified using the Agencourt® Ampure® XP system (Beckman Coulter, Vienna, Austria) and the DNA concentration was determined using a Quant-iT™ PicoGreen® dsDNA Assay (Invitrogen, Darmstadt, Germany). Triplicate PCR reactions for each sample were pooled for sequencing.

Cell extraction and Nycodenz density gradient separation

A 0.5 ml sediment aliquot was fixed with 1 ml 4% paraformaldehyde solution on ice for 5h. Fixed sediment (50-100 mg) was resuspended in 1 ml 1x phosphate buffered saline (PBS) containing 3 mM Na-pyrophosphate and 0.5% Tween 20 and vortexed for 30 minutes at 70% of the maximum speed on a Vortex-Genie 2 (Scientific Industries). After chemical detachment the samples were sonicated on ice at a power setting of 20-25% with a Sonoplus HD 2070 (Bandelin electronic, Berlin, Germany) and then subjected to Nycodenz (1.42 g ml^{-1}) density gradient centrifugation in an ultracentrifuge (Beckman) at 14,000 g for 90 minutes at 4°C using the rotor SWT14i and no deceleration. The top of the Nycodenz phase, which contains most of the microbial cells, was collected with polycarbonate filters (GTTP type, pore size 0.2 µm, Millipore).

Catalyzed reporter deposition fluorescence *in situ* hybridization (CARD-FISH)

CARD-FISH of *Psychrilyobacter* phylotype 4749 and *Desulfobacteraceae* phylotype 2011 was performed using existing probes (Supplementary Table S5) (Greuter *et al.*, 2016). The other target organisms were detected using a two-step CARD-FISH procedure (Wasmund *et al.*, in prep.). In two-step CARD-FISH, two specific unlabeled oligonucleotide probes were hybridized simultaneously for each target organism (Supplementary Table S5). These probes were newly designed or modified from existing probes using the ARB probe design tool (Ludwig *et al.*, 2004). If required, competitor and helper probes were used for increased specificity and target accessibility, respectively. Probes and competitors were evaluated for uniform hybridization behavior with mathFISH (Yilmaz *et al.*, 2011). Each probe applied in the first hybridization of the two-step CARD-FISH procedure consisted of a universal 'adapter' sequence 5'-CCGAATACAAAGCATCAACGACTAGAAAAA-3' and the sequence of the diagnostic probe (Yamaguchi *et al.*, 2015). The second hybridization was performed with a horseradish peroxidase (HRP) labeled detector probe (5'-CTAGTCGTTGATGCTTTGTATTCGG-'3), which is the reverse complement to the 'adapter' sequence. CARD-FISH and two-step CARD-FISH was performed on polycarbonate filters

according to an established protocol (Pernthaler *et al.*, 2002). The embedding of the filter in agarose was omitted in order to facilitate recovery of cells after hybridization. Endogenous peroxidases were inactivated via treatment with 0.1% H_2O_2 in 1x PBS for 2 minutes, followed by permeabilization according to Pernthaler *et al.*, 2002. The cells were then hybridized in 300 µl of standard hybridization buffer (Pernthaler *et al.*, 2002), without salmon sperm DNA and *E.coli* tRNA. The buffers for CARD-FISH and two-step CARD-FISH included 25 or 30% (vol/vol) formamide (Supplementary Table S5) and 1 µl of the probe (50 ng μl^{-1}). For standard CARD-FISH the reaction chamber (1.5 mL microcentrifuge tube) was incubated for 3 h at 46°C (Woebken *et al.*, 2012). For two-step CARD-FISH, the filter was first hybridized for 3 h at 46°C with the specific unlabeled oligonucleotide probe (Supplementary Table S5), followed by a second hybridization that was performed with the detector probe and a formamide concentration of 20% for 2 h. Afterwards, filters were washed and incubated for 15 minutes at room temperature in 1x PBS. Tyramide signal amplification in 1 ml amplification buffer (0.1% Blocking Reagent [wt/vol], 10% dextran sulfate [wt/vol], 2 M NaCl and 1x PBS) supplemented with 10 µL of 0.15% H_2O_2 and 1 µL of OregonGreen® 488 Tyramide solution (1 mg ml^{-1} in dimethylformamide) was performed in the dark for 15 minutes at 46°C. Filters were washed and incubated again for 15 minutes at room temperature in 1x PBS.

Cell separation from filters

Cells were removed from filters by sonication at a power setting of 20-25% on ice followed by vortexing for 30 minutes at 70% of the maximum speed on a Vortex-Genie 2. Suspended cells were pelleted by centrifugation at 10,621 g for 10 minutes and resuspended in 50 µL 1x PBS:ethanol (1:1 vol/vol).

Supplementary Results and Discussion

Differences between ^{12}C-spirulina and ^{13}C-spirulina incubations

The prepared ^{13}C-spirulina and ^{12}C-spirulina solutions were visibly different in regards to color and quality. Concentrations of VFAs produced from ^{13}C-spirulina and ^{12}C-spirulina differed substantially (Figure 1). In particular, the concentrations of most analyzed VFAs in ^{12}C-spirulina incubations at day 0 were considerably higher than in ^{13}C-spirulina incubations (Figure 1). Accordingly, bacterial community responses differed between incubations with HD ^{13}C-spirulina and HD ^{12}C-spirulina (Supplementary Figure S3 and Supplementary Figure S4). Shifts in bacterial community composition and enrichment of responding phylotypes was more pronounced in ^{13}C-spirulina incubations. These observations suggest that the added ^{12}C-spirulina was already partly degraded.

Physiological properties of closest relatives of responsive phylotypes

Psychromonas phylotype 7435 has >99% 16S rRNA identity to different *Psychromonas* species (Supplementary Figure S5) with hydrolytic, fermentative and respiratory capabilities (Kawasaki *et al.*, 2002; Xu, 2003; Riley *et al.*, 2008). *Psychrilyobacter* phylotype 4749 shares 97.4% 16S rRNA identity with *P. atlanticus*, which ferments sugars and amino acids to acetate, butyrate, propionate and other products (Zhao *et al.*, 2009). *P. atlanticus* was shown to metabolize ^{13}C-spirulina in a previous stable isotope probing experiment with temperate tidal flat sediment (Graue *et al.*, 2012). Members of this genus/species might thus have a more ubiquitous role in complex OM degradation in marine sediments. The closest relatives of *Marinifilum* phylotype 4400, *M. fragile* and *M. flexuosum* (97.5% 16S rRNA identity) (Supplementary Figure S5), ferment sugars such as glucose to acetate and propionate (Na *et al.*, 2009; Ruvira *et al.*, 2013). *Colwellia* phylotype 7234 had 100% 16S rRNA sequence identity to *C. rossensis*, which degrades various carbohydrates, fatty acids and amino acids (Techtmann *et al.*, 2016). *Marinilabiliaceae* phylotype 9869 and *Clostridiales* phylotype 1452 have less than 95% 16S rRNA identity

to the next related, fermenting *Saccharicrinis* and *Fusibacter* species, respectively (Supplementary Figure S5), and thus could represent novel genera (Yarza *et al.*, 2014).

Supplementary References

Berger, S.A., Krompass, D., and Stamatakis, A. (2011) Performance, accuracy, and Web server for evolutionary placement of short sequence reads under maximum likelihood. *Syst. Biol.* **60**: 291–302.

Berry, D., Ben Mahfoudh, K., Wagner, M., and Loy, A. (2011) Barcoded primers used in multiplex amplicon pyrosequencing bias amplification. *Appl. Environ. Microbiol.* **77**: 7846–7849.

Berry, D., Schwab, C., Milinovich, G., Reichert, J., Ben Mahfoudh, K., Decker, T., *et al.* (2012) Phylotype-level 16S rRNA analysis reveals new bacterial indicators of health state in acute murine colitis. *ISME J.* **6**: 2091–2106.

Daly, K. and Shirazi-Beechey, S.P. (2003) Design and evaluation of group-specific oligonucleotide probes for quantitative analysis of intestinal ecosystems: their application to assessment of equine colonic microflora. *FEMS Microbiol. Ecol.* **44**: 243–252.

Díez-Vives, C., Gasol, J.M., and Acinas, S.G. (2012) Evaluation of marine bacteroidetes-specific primers for microbial diversity and dynamics studies. *Microb. Ecol.* **64**: 1047–1055.

Graue, J., Engelen, B., and Cypionka, H. (2012) Degradation of cyanobacterial biomass in anoxic tidal-flat sediments: a microcosm study of metabolic processes and community changes. *ISME J.* **6**: 660–669.

Greuter, D., Loy, A., Horn, M., and Rattei, T. (2016) probeBase--an online resource for rRNA-targeted oligonucleotide probes and primers: new features 2016. *Nucleic Acids Res.* **44**: D586–9.

Helgeson, H.C. (1969) Thermodynamics of hydrothermal systems at elevated temperatures and pressures. *Am. J. Sci.* **267**: 729–804.

Helgeson, H.C., Kirkham, D.H., and Flowers, G.C. (1981) Theoretical prediction of the thermodynamic

behavior of aqueous electrolytes by high pressures and temperatures; IV, Calculation of activity coefficients, osmotic coefficients, and apparent molal and standard and relative partial molal properties to 600 degrees C and 5kb. *Am. J. Sci.* **281**: 1249–1516.

Hristova, K.R., Mau, M., Zheng, D., Aminov, R.I., Mackie, R.I., Gaskins, H.R., and Raskin, L. (2000) *Desulfotomaculum* genus- and subgenus-specific 16S rRNA hybridization probes for environmental studies. *Environ. Microbiol.* **2**: 143–159.

Johnson, J.W., Oelkers, E.H., and and Helgeson, H.C. (1992) SUPCRT92: A software package for calculating the standard molal thermodynamic properties of minerals, gases, aqueous species, and reactions from 1 to 5000 bar and 0 to 1000°C. *Comput. Geosci.* **18**: 899–947.

Jørgensen, B.B., Dunker, R., Grünke, S., and Røy, H. (2010) Filamentous sulfur bacteria, *Beggiatoa* spp., in arctic marine sediments (Svalbard, 79 degrees N). *FEMS Microbiol. Ecol.* **73**: 500–513.

Kawasaki, K., Matsuyama, H., Yumoto, I., Hishinuma, M., Nodasaka, Y., and Nogi, Y. (2002) *Psychromonas marina* sp. nov., a novel halophilic, facultatively psychrophilic bacterium isolated from the coast of the Okhotsk Sea. *Int. J. Syst. Evol. Microbiol.* **52**: 1455–1459.

Lin, X., Wakeham, S.G., Putnam, I.F., Astor, Y.M., Scranton, M.I., Chistoserdov, A.Y., and Taylor, G.T. (2006) Comparison of vertical distributions of prokaryotic assemblages in the anoxic Cariaco Basin and Black Sea by use of fluorescence in situ hybridization. *Appl. Environ. Microbiol.* **72**: 2679–2690.

Ludwig, W., Strunk, O., Westram, R., Richter, L., Meier, H., Yadhukumar, *et al.* (2004) ARB: a software environment for sequence data. *Nucleic Acids Res.* **32**: 1363–1371.

Lueders, T., Pommerenke, B., and Friedrich, M.W. (2004) Stable-isotope probing of microorganisms thriving at thermodynamic limits: syntrophic propionate oxidation in flooded soil. *Appl. Environ. Microbiol.* **70**: 5778–5786.

Na, H., Kim, S., Moon, E.Y., and Chun, J. (2009) Marinifilum fragile gen. nov., sp. nov., isolated from tidal flat sediment. *Int. J. Syst. Evol. Microbiol.* **59**: 2241–2246.

Pernthaler, A., Pernthaler, J., and Amann, R. (2002) Fluorescence in situ hybridization and catalyzed reporter deposition for the identification of marine bacteria. *Appl. Environ. Microbiol.* **68**: 3094–3101.

Price, M.N., Dehal, P.S., and Arkin, A.P. (2009) FastTree: computing large minimum evolution trees with profiles instead of a distance matrix. *Mol. Biol. Evol.* **26**: 1641–1650.

Pruesse, E., Peplies, J., and Glöckner, F.O. (2012) SINA: accurate high-throughput multiple sequence alignment of ribosomal RNA genes. *Bioinformatics* **28**: 1823–1829.

Quast, C., Pruesse, E., Yilmaz, P., Gerken, J., Schweer, T., Yarza, P., *et al.* (2013) The SILVA ribosomal RNA gene database project: improved data processing and web-based tools. *Nucleic Acids Res.* **41**: D590–6.

Riley, M., Staley, J.T., Danchin, A., Wang, T.Z., Brettin, T.S., Hauser, L.J., *et al.* (2008) Genomics of an extreme psychrophile, *Psychromonas ingrahamii*. *BMC Genomics* **9**: 210.

Ruvira, M.A., Lucena, T., Pujalte, M.J., Arahal, D.R., and Carmen Macián, M. (2013) *Marinifilum flexuosum* sp. nov., a new *Bacteroidetes* isolated from coastal Mediterranean Sea water and emended description of the genus *Marinifilum* Na *et al.*, 2009. *Syst. Appl. Microbiol.* **36**: 155–159.

Shock, E.L. (1995) Organic acids in hydrothermal solutions: standard molal thermodynamic properties of carboxylic acids and estimates of dissociation constants at high temperatures and pressures. *Am. J. Sci.* **295**: 496–580.

Shock, E.L. and Helgeson, H.C. (1988) Calculation of the thermodynamic and transport properties of aqueous species at high pressures and temperatures: Correlation algorithms for ionic species and equation of state predictions to 5 kb and 1000°C. *Geochim. Cosmochim. Acta* **52**: 2009–2036.

Shock, E.L. and Helgeson, H.C. (1990) Calculation of the thermodynamic and transport properties of aqueous species at high pressures and temperatures: Standard partial molal properties of organic species. *Geochim. Cosmochim. Acta* **54**: 915–945.

Snaidr, J., Fuchs, B., Wallner, G., Wagner, M., Schleifer, K.H., and Amann, R. (1999) Phylogeny and in

situ identification of a morphologically conspicuous bacterium, Candidatus *Magnospira bakii*, present at very low frequency in activated sludge. *Environ. Microbiol.* **1**: 125–135.

Stamatakis, A. (2014) RAxML version 8: a tool for phylogenetic analysis and post-analysis of large phylogenies. *Bioinformatics* **30**: 1312–1313.

Tanger, J.C. and Helgeson, H.C. (1988) Calculation of the thermodynamic and transport properties of aqueous species at high pressures and temperatures; revised equations of state for the standard partial molal properties of ions and electrolytes. *Am. J. Sci.* **288**: 19–98.

Techtmann, S.M., Fitzgerald, K.S., Stelling, S.C., Joyner, D.C., Uttukar, S.M., Harris, A.P., *et al.* (2016) *Colwellia psychrerythraea* Strains from distant deep sea basins show adaptation to local conditions. *Front. Environ. Sci. Eng. China* **4**: 33.

Vandieken, V., Finke, N., and Jørgensen, B.B. (2006) Pathways of carbon oxidation in an Arctic fjord sediment (Svalbard) and isolation of psychrophilic and psychrotolerant Fe(III)-reducing bacteria. *Mar. Ecol. Prog. Ser.* **322**: 29–41.

Wehrmann, L.M., Formolo, M.J., Owens, J.D., Raiswell, R., Ferdelman, T.G., Riedinger, N., and Lyons, T.W. (2014) Iron and manganese speciation and cycling in glacially influenced high-latitude fjord sediments (West Spitsbergen, Svalbard): Evidence for a benthic recycling-transport mechanism. *Geochim. Cosmochim. Acta* **141**: 628–655.

Wehrmann, L.M., Riedinger, N., Brunner, B., Kamyshny, A., Hubert, C.R.J., Herbert, L.C., *et al.* (2017) Iron-controlled oxidative sulfur cycling recorded in the distribution and isotopic composition of sulfur species in glacially influenced fjord sediments of west Svalbard. *Chem. Geol.* **466**: 678–695.

Woebken, D., Burow, L.C., Prufert-Bebout, L., Bebout, B.M., Hoehler, T.M., Pett-Ridge, J., *et al.* (2012) Identification of a novel cyanobacterial group as active diazotrophs in a coastal microbial mat using NanoSIMS analysis. *ISME J.* **6**: 1427–1439.

Xu, Y. (2003) Psychromonas profunda sp. nov., a psychropiezophilic bacterium from deep Atlantic sediments. *Int. J. Syst. Evol. Microbiol.* **53**: 527–532.

Yamaguchi, T., Kawakami, S., Hatamoto, M., Imachi, H., Takahashi, M., Araki, N., *et al.* (2015) In situ DNA-hybridization chain reaction (HCR): a facilitated in situ HCR system for the detection of environmental microorganisms. *Environ. Microbiol.* **17**: 2532–2541.

Yarza, P., Yilmaz, P., Pruesse, E., Glöckner, F.O., Ludwig, W., Schleifer, K.-H., *et al.* (2014) Uniting the classification of cultured and uncultured bacteria and archaea using 16S rRNA gene sequences. *Nat. Rev. Microbiol.* **12**: 635–645.

Yilmaz, L.S., Parnerkar, S., and Noguera, D.R. (2011) mathFISH, a web tool that uses thermodynamics-based mathematical models for in silico evaluation of oligonucleotide probes for fluorescence in situ hybridization. *Appl. Environ. Microbiol.* **77**: 1118–1122.

Zhao, J.-S., Manno, D., and Hawari, J. (2009) *Psychrilyobacter atlanticus* gen. nov., sp. nov., a marine member of the phylum *Fusobacteria* that produces H2 and degrades nitramine explosives under low temperature conditions. *Int. J. Syst. Evol. Microbiol.* **59**: 491–497.

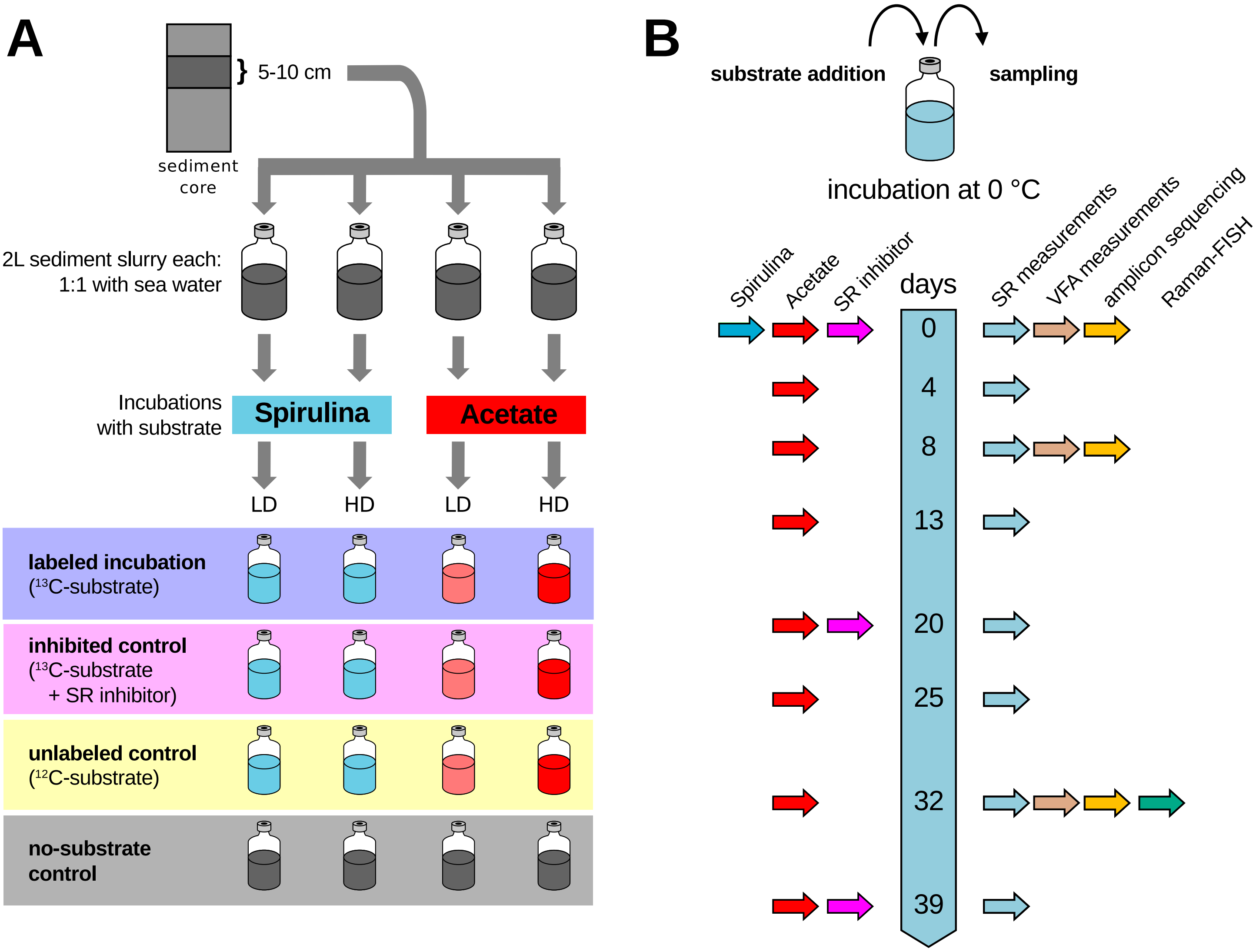

Supplementary Figure S1. Experimental overview. A. Setup of anoxic sediment incubations. B. Timeline for substrate additions and sampling. LD, low substrate dose (50 µg ml^{-1} spirulina and 50 µM acetate). HD, high substrate dose (1 mg ml^{-1} spirulina and 1 mM acetate). SR-inhibitor, the sulfate reduction inhibitor molybdate.

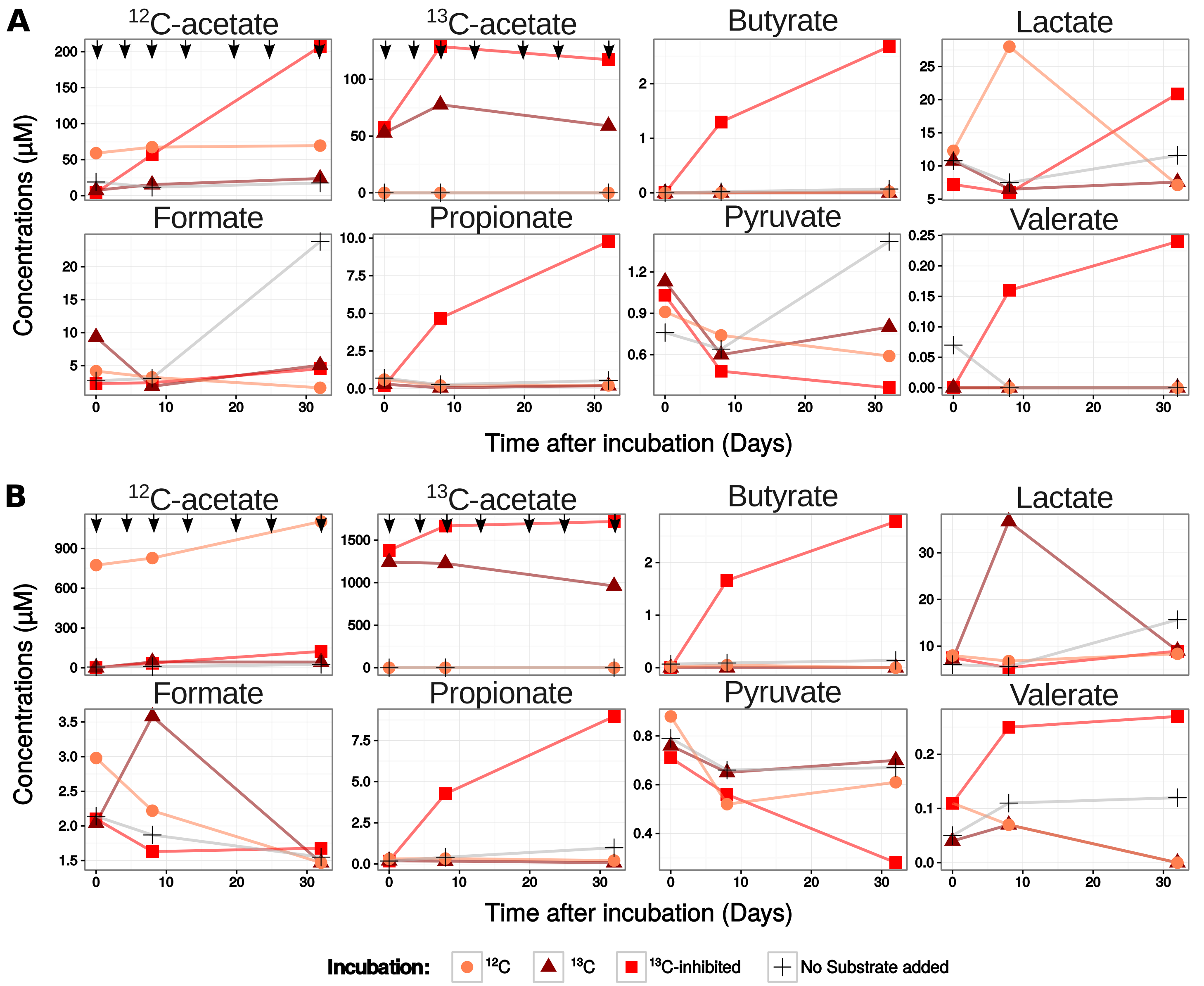

Supplementary Figure S2. Volatile fatty acids concentrations in acetate incubations. Concentrations of volatile fatty acids in (A) LD and (B) HD incubations with acetate. ^{13}C-inhibited, sediment incubations with ^{13}C-substrate and molybdate. Note that acetate was periodically added (black arrows) right before the measurement. LD, low dose (50 µM) of acetate. HD, high dose (1 mM) of acetate. Note that the scales are different for each treatment and each VFA.

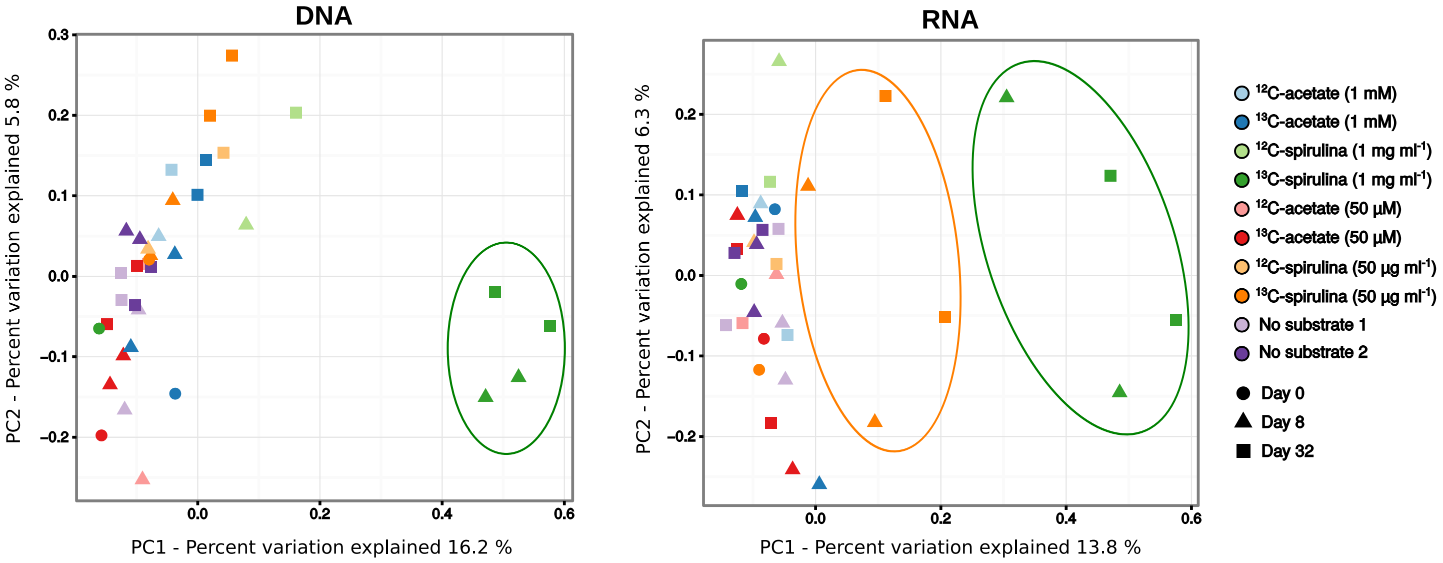

Supplementary Figure S3. Principal coordinates analysis of bacterial beta-diversity in anoxic sediment incubations. The analyses were based on Bray-Curtis dissimilarities of relative 16S rRNA gene (DNA) and transcript (RNA) abundances of bacterial phylotypes. Sample color indicates type and concentration of added substrate. Sample shape indicates the day of sampling.

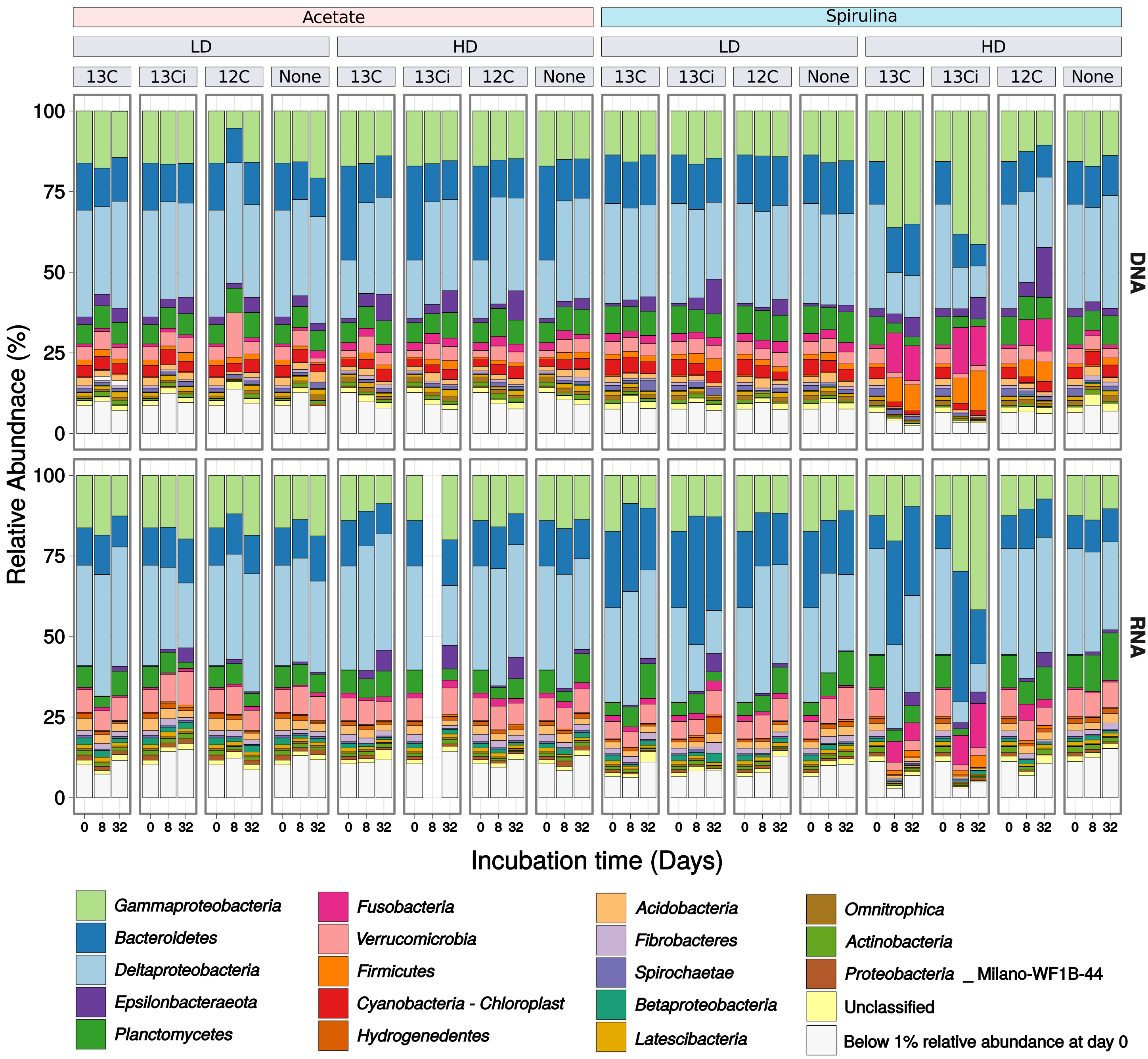

Supplementary Figure S4. Bacterial community dynamics in spirulina and acetate amended anoxic sediment incubations. Only phyla/classes with a relative 16S rRNA gene (DNA) and/or transcript (RNA) abundance of ≥1% at day 0 are indicated. 13C., sediment incubations with ^{13}C-substrate. 13Ci., sediment incubations with ^{13}C-substrate and molybdate. 12C., sediment incubations with ^{12}C-substrate. None., no-substrate control. LD, low substrate dose (50 µg ml^{-1} spirulina or 50 µM acetate). HD, high substrate dose (1 mg ml^{-1} spirulina or 1 mM acetate).

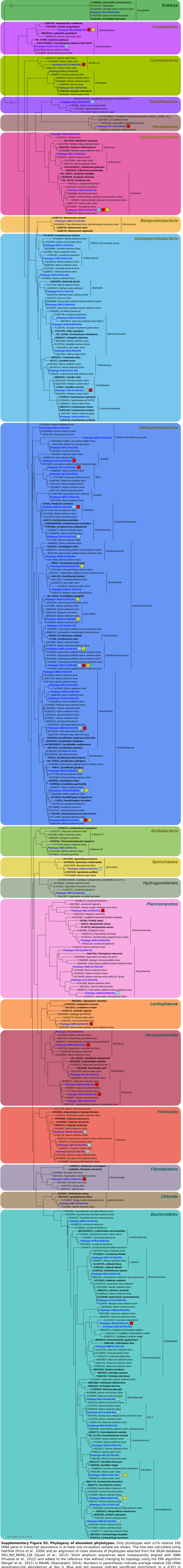

Supplementary Figure S5. Phylogeny of abundant phylotypes. Only phylotypes with ≥1% relative 16S rRNA gene or transcript abundance in at least one incubation sample are shown. The tree was calculated using FastTree (Price *et al.*, 2009) and an alignment of close relatives of phylotypes selected from the SILVA database SSU_Ref_NR99_128 (Quast *et al.*, 2013). Short amplicon sequences were subsequently aligned with SINA (Pruesse *et al.*, 2012) and added to the reference tree without changing its topology using the EPA algorithm (Berger *et al.*, 2011) in RAxML (Stamatakis, 2014). Numbers in parentheses indicate average relative 16S rRNA gene/transcript abundances at day 0. Blue and red squares indicate significant enrichment ($p \leq 0.01$) in spirulina and acetate incubations, respectively, compared to the no substrate control. Numbers within the squares give the number of samples (of 24 spirulina and 22 acetate incubation samples in total) in which the phylotype was significantly enriched. Yellow squares indicate sulfate-reduction-associated phylotypes, numbers in the square indicate numbers of samples (of 15 in total) in which the phylotype was significantly enriched in uninhibited incubations with substrate compared to molybdate-inhibited controls.

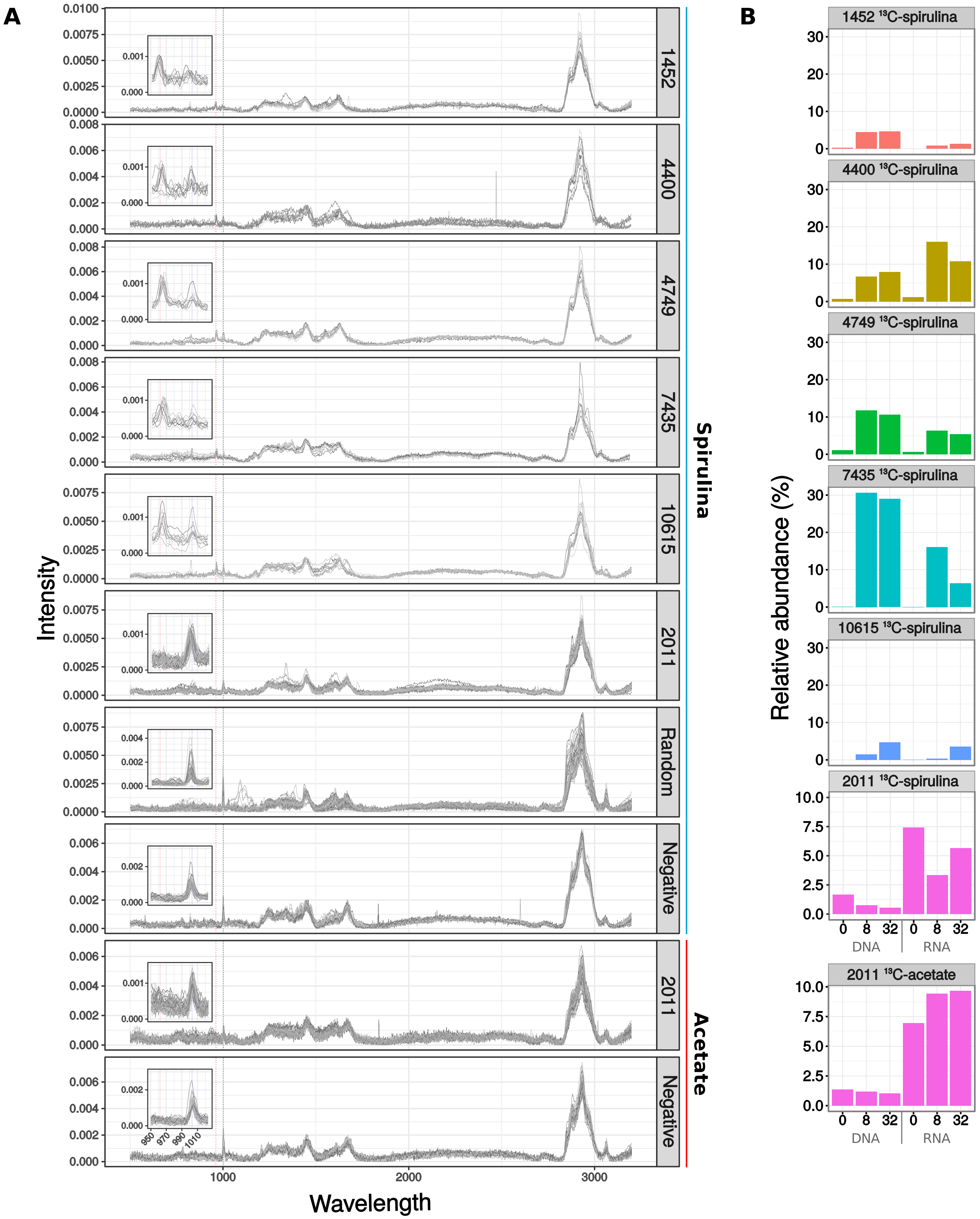

Supplementary Figure S6. Raman spectra of responsive phylotypes and their relative sequence abundance. (A) Overlays of single cell Raman spectra are displayed for the CARD-FISH-probe-labelled phylotypes in ^{13}C-spirulina (1 mg ml^{-1}) or ^{13}C-acetate (1 mM) incubations. *Clostridiales* phylotype 1452 (*Firmicutes*), *Marinifilum* phylotype 4400 (*Bacteroidetes*), *Psychrilyobacter* phylotype 4749 (*Fusobacteria*), *Psychromonas* phylotype 7435 (*Gammaproteobacteria*), *Arcobacter* phylotype 10615 (*Epsilonbacteraeota*), and *Desulfobacteraceae* phylotype 2011 (*Deltaproteobacteria*). Random, Raman spectra from randomly selected cells in the ^{13}C-spirulina (1 mg ml^{-1}) incubations. Negative ^{13}C-spirulina, Raman spectra from randomly selected cells from the ^{12}C-spirulina incubations. Negative ^{13}C-acetate, Raman spectra from cells of *Desulfobacteraceae* phylotype 2011 (*Deltaproteobacteria*) from incubations with ^{12}C-acetate at day 0. Insets show enlarged Raman spectrum regions containing ^{13}C-phenylalanine (wavelength of 960-970 cm^{-1}) and ^{12}C-phenylalanine (wavelength of 1000-1005 cm^{-1}) peaks. (B) Relative 16S rRNA gene and transcript abundance of CARD-FISH targeted cell populations in the respective substrate supplemented sediment incubation.

Supplementary Table S1. Relative abundances of phylotypes with ≥1% of all bacterial 16S rRNA genes or transcripts at day 0.

Phylotype	Taxonomy (phylum; class; order; family; genus)	DNA	RNA
4247	*Bacteroidetes; Bacteroidia; Bacteroidales; Marinilabiliaceae*	0.7%	1.6%
4400	*Bacteroidetes; Bacteroidia; Bacteroidales; Marinilabiliaceae; Marinifilum*	1.5%	2.7%
4270	*Bacteroidetes; Bacteroidia;* BD2-2	1.6%	0.8%
10972	*Bacteroidetes; Flavobacteriia; Flavobacteriales; Flavobacteriaceae; Lutibacter*	2.7%	0.0%
1270	*Bacteroidetes; Flavobacteriia; Flavobacteriales; Flavobacteriaceae; Lutimonas*	2.8%	0.0%
8760	*Cyanobacteria; Chloroplast*	2.4%	0.0%
4749	*Fusobacteria; Fusobacteriia; Fusobacteriales; Fusobacteriaceae; Psychrilyobacter*	1.7%	1.1%
44	*Planctomycetes; Phycisphaerae; Phycisphaerales*	1.3%	0.2%
11461	*Proteobacteria; Betaproteobacteria; Nitrosomonadales; Nitrosomonadaceae; Nitrosomonas*	0.1%	1.6%
10184	*Proteobacteria; Deltaproteobacteria; Desulfobacterales; Desulfobacteraceae;* SEEP-SRB1	1.4%	1.2%
2011	*Proteobacteria; Deltaproteobacteria; Desulfobacterales; Desulfobacteraceae;* Sva0081 sediment group	1.3%	6.8%
11380	*Proteobacteria; Deltaproteobacteria; Desulfobacterales; Desulfobacteraceae;* Sva0081 sediment group	1.1%	2.4%
4982	*Proteobacteria; Deltaproteobacteria; Desulfobacterales; Desulfobulbaceae*	0.7%	1.8%
6023	*Proteobacteria; Deltaproteobacteria; Desulfobacterales; Desulfobulbaceae; Desulfobulbus*	3.1%	1.0%
10263	*Proteobacteria; Deltaproteobacteria; Desulfobacterales; Desulfobulbaceae; Desulfobulbus*	0.2%	1.8%
3714	*Proteobacteria; Deltaproteobacteria; Desulfuromonadales;* Sva1033	2.6%	0.1%
2526	*Proteobacteria; Deltaproteobacteria;* Sva0485	1.5%	0.2%
2799	*Proteobacteria; Gammaproteobacteria*	1.0%	3.2%
2228	*Proteobacteria; Gammaproteobacteria; Alteromonadales; Alteromonadaceae;* BD2-7	1.3%	0.3%
7234	*Proteobacteria; Gammaproteobacteria; Alteromonadales; Colwelliaceae; Colwellia*	2.2%	0.2%
9245	*Proteobacteria; Gammaproteobacteria;* BD7-8 marine group	1.8%	0.9%
62	*Proteobacteria; Gammaproteobacteria; Gammaproteobacteria Incertae Sedis*	3.1%	3.1%
6148	*Proteobacteria; Gammaproteobacteria; Thiotrichales; Thiotrichaceae*	0.4%	1.2%
9135	*Proteobacteria; Gammaproteobacteria; Xanthomonadales;* JTB255 marine benthic group	1.5%	0.2%
6150	*Proteobacteria;* Milano-WF1B-44	0.2%	1.2%

Supplementary Table S2. Read number, coverage, and alpha-diversity of bacterial 16S rRNA gene and transcript libraries.

Incubation	Sample type	High quality reads			Observed phylotypes (all seqs)			Good's coverage (all seqs)			Observed phylotypes (3250 seqs per library)			Chao1 richness (3250 seqs per library)			Simpson diversity index (3250 seqs per library)			Shannon diversity index (3250 seqs per library)			Equitability (3250 seqs per library)		
		day 0[a]	day 8	day 32	day 0	day 8	day 32	day 0	day 8	day 32	day 0	day 8	day 32	day 0	day 8	day 32	day 0	day 8	day 32	day 0	day 8	day 32	day 0	day 8	day 32
**50 µM ^{13}C acetate**	DNA	5523	3770	4023	1083	847	861	0.899	0.884	0.899	818	785	777	1589	1447	1306	0.991	0.991	0.992	8.17	8.17	8.20	0.844	0.850	0.854
**Control (50 µM ^{12}C acetate)**	DNA		262	4078		124	853		0.698	0.895		-	759		-	1364		-	0.991		-	8.10		-	0.846
**Control (50 µM ^{13}C acetate + inhibitor)**	DNA		4277	4468		949	891		0.884	0.901		820	759		1558	1384		0.991	0.991		8.21	8.08		0.848	0.845
**Control (No Substrate)**	DNA		4041	351		887	154		0.886	0.732		791	-		1494	-		0.991	-		8.18	-		0.849	-
**1 mM ^{13}C acetate**	DNA	3282	4281	3788	643	953	798	0.903	0.880	0.895	640	819	740	1104	1631	1294	0.969	0.992	0.988	7.18	8.24	7.94	0.771	0.852	0.833
**Control (1 mM ^{12}C acetate)**	DNA		4680	4360		928	853		0.907	0.904		777	737		1334	1313		0.991	0.988		8.16	7.94		0.850	0.834
**Control (1 mM ^{13}C acetate + inhibitor)**	DNA		4687	5687		934	1022		0.905	0.912		779	766		1375	1417		0.991	0.989		8.12	7.98		0.846	0.833
**Control (No Substrate)**	DNA		6035	4145		1151	910		0.903	0.894		827	805		1621	1373		0.991	0.992		8.20	8.27		0.847	0.857
**50 µg/l ^{13}C spirulina**	DNA	6013	5141	5073	1065	1034	943	0.919	0.897	0.907	786	809	748	1395	1554	1402	0.989	0.990	0.990	8.04	8.07	7.98	0.836	0.835	0.836
**Control (50 µg/l ^{12}C spirulina)**	DNA		1071	5181		295	972		0.855	0.911		-	766		-	1380		-	0.990		-	8.04		-	0.839
**Control (50 µg/l ^{13}C spirulina + inhibitor)**	DNA		5017	4510		947	817		0.909	0.912		762	692		1359	1215		0.990	0.983		8.05	7.63		0.840	0.809
**Control (No Substrate)**	DNA		4826	4992		980	996		0.897	0.895		792	791		1476	1552		0.989	0.990		8.00	8.04		0.831	0.836
**1 µM ^{13}C spirulina**	DNA	4251	5213	5984	869	600	597	0.902	0.940	0.949	762	466	428	1321	909	856	0.991	0.883	0.892	8.14	5.46	5.36	0.851	0.616	0.613
**Control (1 µM ^{12}C spirulina)**	DNA		4342	5744		795	905		0.914	0.920		690	669		1180	1273		0.987	0.968		7.82	7.21		0.829	0.768
**Control (1 µM ^{13}C spirulina + inhibitor)**	DNA		3802	4486		464	461		0.936	0.945		427	387		776	738		0.869	0.845		5.22	4.84		0.597	0.563
**Control (No Substrate)**	DNA		700	4873		259	973		0.794	0.898		-	790		-	1478		-	0.991		-	8.15		-	0.847
**50 µM ^{13}C acetate**	RNA	5572	8826	4540	1057	1283	967	0.912	0.935	0.892	813	790	813	1436	1424	1478	0.989	0.985	0.985	8.18	7.91	8.04	0.846	0.822	0.832
**Control (50 µM ^{12}C acetate)[b]**	RNA		n.d.	5342		n.d.	967		n.d.	0.912		n.d.	751		n.d.	1385		n.d.	0.984		n.d.	7.75		n.d.	0.812
**Control (50 µM ^{13}C acetate + inhibitor)**	RNA		6351	4708		1266	979		0.907	0.903		906	818		1659	1414		0.992	0.991		8.54	8.27		0.869	0.855
**Control (No Substrate)**	RNA		5713	5386		1159	1090		0.904	0.902		872	845		1569	1541		0.990	0.991		8.33	8.29		0.852	0.852
**1 mM ^{13}C acetate**	RNA	5940	5890	4102	1061	1028	852	0.916	0.919	0.897	788	765	759	1400	1377	1323	0.988	0.983	0.982	8.11	7.86	7.81	0.843	0.821	0.816
**Control (1 mM ^{12}C acetate)**	RNA		6249	3298		1116	734		0.914	0.890		799	729		1481	1213		0.987	0.985		7.99	7.86		0.829	0.826
**Control (1 mM ^{13}C acetate + inhibitor)[b]**	RNA		n.d.	4327		n.d.	819		n.d.	0.909		n.d.	712		n.d.	1240		n.d.	0.988		n.d.	7.88		n.d.	0.832
**Control (No Substrate)**	RNA		6194	4552		1048	997		0.917	0.893		750	843		1387	1485		0.984	0.989		7.76	8.26		0.813	0.850
**50 µg/l ^{13}C spirulina**	RNA	7325	5489	1443	1141	899	439	0.929	0.922	0.838	760	686	-	1400	1257	-	0.983	0.973	-	7.68	7.29	-	0.803	0.774	-
**Control (50 µg/l ^{12}C spirulina)**	RNA		6405	5298		974	1017		0.931	0.911		702	802		1238	1379		0.982	0.989		7.59	8.12		0.803	0.842
**Control (50 µg/l ^{13}C spirulina + inhibitor)**	RNA		6797	210		1008	108		0.933	0.676		707	-		1249	-		0.965	-		7.34	-		0.776	-
**Control (No Substrate)**	RNA		5787	4317		1074	949		0.912	0.892		804	822		1448	1462		0.984	0.989		7.94	8.22		0.823	0.849
**1 µM ^{13}C spirulina**	RNA	4822	5901	4807	1013	630	715	0.899	0.949	0.931	829	465	589	1469	854	1013	0.989	0.928	0.968	8.25	5.68	6.86	0.851	0.641	0.745
**Control (1 µM ^{12}C spirulina)**	RNA		6835	5204		1017	1008		0.933	0.904		708	790		1252	1476		0.982	0.982		7.65	7.91		0.808	0.821
**Control (1 µM ^{13}C spirulina + inhibitor)**	RNA		5820	3828		479	409		0.960	0.948		353	377		664	636		0.848	0.883		4.51	5.21		0.533	0.608
**Control (No Substrate)**	RNA		5431	5593		1035	1115		0.916	0.913		812	865		1355	1455		0.987	0.991		8.17	8.52		0.846	0.873

[a]DNA/RNA samples from day 0 were pooled prior to PCR amplification.
[b]n.d., no data due to an error in the barcode sequence

Supplementary Table S3. Data for calculation of *in situ* Gibbs energy of acetoclastic sulfate reduction.

Parameter	Value
Reaction	$C_2H_3O_2^- + SO_4^{2-} \rightarrow 2\ HCO_3^- + HS^-$
T [K][1]	273.55
p [bar]	1
Acetate [µM][2]	12
Sulfate [mM][3]	26.5
HCO_3^- [mM][4]	3
HS^- [µM][3]	2
DG_0 (T,p) [kJ mol^{-1}]	-48.51
DG_r (T,p) [kJ mol^{-1}]	-93.2

[1]data taken from Jørgensen *et al.*, 2010
[2]This study. Average concentration in unamended sediment incubations
[3]data taken from Wehrmann *et al.*, 2017
[4]data taken from Vandieken *et al.*, 2006

Supplementary Table S4. Thermodynamic properties used for the calculation of standard state Gibbs energy change of reaction (DG$_0$ (*T*,*p*)). Using the SUPCRT92 software (Johnson *et al.*, 1992). References: a) Schock, 1995 and b) Schock and Helgeson, 1988.

**Species**	**Gamma***	**G_f^0**	**H_f^0**	**S**	**V**	**C_p**	**Reference**
	T=275 K I=0.7	[kJ mol^{-1}]	[kJ mol^{-1}]	[J mol^{-1} K^{-}]	[cm^3 mol^{-1}]	[J mol^{-1} K^{-}]	
$C_2H_3O_2$	0.67	-369.32	486.1	86.19	40.1	26.99	a)
SO_4^{2-}	0.17	-744.46	-909.6	18.83	12.92	-266.1	b)
HCO_3^-	0.67	-586.94	-689.93	98.45	24.21	34.85	b)
HS^-	0.63	11.97	-16.11	68.2	20.35	-93.93	b)

*calculated using the software Geochemists Workbench ® (www.gwb.com).

Supplementary Table S5. 16S rRNA-targeted FISH probes.

Probe name	Probe full name[a]	ΔG[b] [kcal/mol]	Probe sequence (5'-3')	Form-amide [%]	RDP II probe match[c]			Non-target phylotypes[d]	Reference
					Perfectly matched target group / phylotype	Coverage (%)	Non-target group hits		
PSY1446	S-G-PSY-1446-a-A-19	27.1	TCGCCATCCCCGAAAGGTT	25	*Psychromonas* (incl. phylotype 7435)	46.4	0	3	This study
PSY1421	S-G-PSY-1421-a-A-18	24.4	TGACTTCTGGTGCAGCCC	25	*Psychromonas* (incl. phylotype 7435)	95.8	138	2	This study
MARI204 (modified Mkc219)	S-G-MARI-0204-a-A-20	24.2	CATGCCCATCTTATACCACC	25	*Marinifilum* (incl. phylotype 4400)	61.7	442	1	Modified from Snaidr *et al.*, 1999
MARI867 (modified CF418)	S-G-MARI-0867-a-A-21	25.9	CCTTTGAGTTTCATTCTTGCG	25	*Marinifilum* (incl. phylotype 4400)	14.5	115229	7	Modified from Díez-Vives *et al.*, 2012
Competitor for MARI867	cS-*-MARI-0867-a-A-23	29.8	CCTTTGAGTTTCAGTCTTGCGA	25	-	-	-	-	This study
CLOS227 (modified	S-O-CLOS-0227-a-A-23	31.1	AGCTAATCAGACGCGAGTCCA	25	*Clostridiales* (incl. phylotype 1452)	1.1	108	0	Modified from Hristova *et al.*, 2000
Competitor for CLOS227	cS-*-CLOS-0228-a-A-21	29.5	GCTAATGGTACGCGGGCTCAT	25	-	-	-	-	This study
Competitor for CLOS227	cS-*-CLOS-0228-a-A-23	31.4	GCTAATGGTACGCAGGCTCAT	25	-	-	-	-	This study
CLOS1426 (modified III1421)	S-O-CLOS-1426-a-A-17	25.9	TCACTGGCTTCGGGCGC	25	*Clostridiales* (incl. phylotype 1452)	0.4	18	2	Modified from Daly and Shirazi-Beechey, 2003
ARCO1431 (modified	S-G-ARCO-1431-a-A-15	21.3	AGCATCCCCGCTTCG	25	*Arcobacter* (incl. phylotype 10615)	82.3	464	2	Modified from Snaidr *et al.*, 1999
ARCO548 (modified	S-G-ARCO-0548-a-A-20	26.7	CCAGTGATTCCGAGTAACGC	25	*Arcobacter* (incl. phylotype 10615)	93	13604	2	Modified from Lin *et al.*, 2006
Competitor for ARCO548	cS-*-ARCO-0548-a-A-19	24.6	CAGTGATTCCGGATAACGC	25	-	-	-	-	This study
Fuso1	S-O-FUSO-0387-a-A-20	24.9	GCACACAGAATTGCTGGATC	25	*Fusobacteria* (incl. *Psychrilyobacter* phylotype 4749)	93.3	35	0	Sanguin *et al.*, 2006
DSS1431	S-G-DSS-1431-a-A-18	23.6	GGTTTGCCCAACGACTTC	30	*Desulfobacteraceae* (Sva0081 group incl. phylotype 2011)	2	12	2	Dyksma *et al.*, in prep.
Competitor for DSS1431	cS-G-DSS-1431-a-A-18	22.2	GGTTTGCCCAACAACTTC	30	-	-	-	-	Dyksma *et al.*, in prep.
Competitor for DSS1431	cS-G-DSS-1431-a-A-18	21.7	GGTTAGCCCAACAACTTC	30	-	-	-	-	Dyksma *et al.*, in prep.
Competitor for DSS1431	cS-G-DSS-1431-a-A-18	24.5	GGTTCGCCCACCAACTTC	30	-	-	-	-	Dyksma *et al.*, in prep.
Competitor for DSS1431	cS-G-DSS-1431-a-A-18	21.6	AGTTTGCCCAACAACTTC	30	-	-	-	-	Dyksma *et al.*, in prep.
Competitor for DSS1431	cS-G-DSS-1431-a-A-18	23.1	GGTTGGCCCAACAACTTC	30	-	-	-	-	Dyksma *et al.*, in prep.
Helper for DSS1431	hS-G-DSS-1431-a-A-18	26.7	TGGTACAACCAACTCTCATGG	30	-	-	-	-	Dyksma *et al.*, in prep.
Helper for DSS1431	hS-G-DSS-1431-a-A-18	31.6	TTAGGCGCCTGCATCCCCGAA	30	-	-	-	-	Dyksma *et al.*, in prep.
Helper for DSS1431	hS-G-DSS-1431-a-A-18	29.9	TTAGGCGCCTGCATCCTGTAA	30	-	-	-	-	Dyksma *et al.*, in prep.

[a]According to Alm *et al.* (1996)
[b]ΔG: Free energy for perfect match hybrid was predicted with Oligocalc (Sanguin *et al.*, 2006)
[c]RDP II probe match was performed with database release 11, Update 5 (September 30, 2016) containing 3,356,809 16S rRNA sequences. The search for each probe was restricted to sequences of good quality with data in the respective probe binding region. Coverage is defined as the percentage of sequences in the RDP II target taxon with a full match to the probe sequence. The number of non-target hits indicates the total number of perfectly matching sequences outside the respective RDP II target taxon.
[d]Number of additional, perfectly-matched phylotypes from this study with >0.1% relative 16S rRNA gene abundance in at least one sample
